# Multifunctional Heterometallic Ir^III^−Au^I^ Probes as Promising Anticancer and Antiangiogenic Agents

**DOI:** 10.1002/chem.202100707

**Published:** 2021-05-29

**Authors:** Marta Redrado, Andrea Benedi, Isabel Marzo, Angel L. García‐Otín, Vanesa Fernández‐Moreira, M. Concepción Gimeno

**Affiliations:** ^1^ Departamento de Química Inorgánica Instituto de Síntesis Química y Catálisis Homogénea (ISQCH) CSIC-Universidad de Zaragoza 50009 Zaragoza Spain; ^2^ Departamento de Bioquímica y Biología Celular Universidad de Zaragoza-CSIC 50009 Zaragoza Spain; ^3^ Unidad de Investigación Traslacional Hospital Universitario Miguel Servet Instituto Aragonés de Ciencias de la Salud (IACS)/ Instituto de Investigación Sanitaria Aragón 50009 Zaragoza Spain

**Keywords:** anti-angiogenesis, cell imaging, gold, heterometallic compounds, iridium, theranostic agents

## Abstract

A new class of emissive cyclometallated Ir^III^−Au^I^ complexes with a bis(diphenylphosphino) methanide bridging ligand was successfully synthesised from the diphosphino complex [Ir(N^C)_2_(dppm)]^+^ (**1**). The different gold ancillary ligand, a triphenylphosphine (**2**), a chloride (**3**) or a thiocytosine (**4**) did not reveal any significant effect on the photophysical properties, which are mainly due to metal‐to‐ligand charge‐transfer (^3^MLCT) transitions based on Ir^III^. However, the Au^I^ fragment, along with the ancillary ligand, seemed crucial for the bioactivity in A549 lung carcinoma cells versus endothelial cells. Both cell types display variable sensitivities to the complexes (IC_50_=0.6–3.5 μM). The apoptotic pathway is activated in all cases, and paraptotic cell death seems to take place at initial stages in A549 cells. Species **2**–**4** showed at least dual lysosomal and mitochondrial biodistribution in A549 cells, with an initial lysosomal localisation and a possible trafficking process between both organelles with time. The bimetallic Ir^III^−Au^I^ complexes disrupted the mitochondrial transmembrane potential in A549 cells and increased reactive oxygen species (ROS) generation and thioredoxin reductase (TrxR) inhibition in comparison with that displayed by the monometallic complex **1**. Angiogenic activity assays performed in endothelial cells revealed the promising antimetastatic potential of **1**, **2** and **4**.

## Graphical abstract

**A golden opportunity**: Cytotoxic and green‐emissive orthometallated Ir^III^‐Au^I^ complexes have been synthesised. For some of the complexes, trafficking between the lysosomes and mitochondria in A549 cells can be suggested. Disruption of the mitochondrial membrane potential was observed as well as an apoptotic cell death mechanism. Angiogenic assays reveal that the complexes have good prospects as antimetastatic agents.

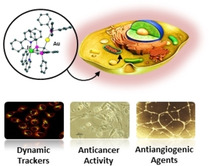

## Introduction

Among the numerous chemotherapeutic drugs, metal‐based anticancer complexes such as cisplatin, carboplatin and oxaliplatin are considered to be the first‐line treatment for many cancer diseases.^[1]^ Despite their proven efficiency, they also present considerable side effects.^[2]^ The nonspecific toxicity together with the generated drug resistance is forcing the development of new metallodrugs that can circumvent such disadvantages. In that line, gold(I) complexes aroused as a great alternative due to the different mechanism of action from that of platinum based drugs (inhibition of thioredoxin reductase TrRx or other thiol enzymes versus DNA binding, respectively) that could allow to reach a wider range of cancer tumours or the treatment of those already resistant to platinum drugs. In fact, auranofin, a gold(I) based‐drug approved by the FDA for rheumatoid arthritis in 1985,^[3]^ is currently at the stage of clinical trials for ovarian cancer treatment.^[4]^ Therefore, an increasing amount of research is devoted to the screening of many gold(I) complexes as cancer drugs. Among them, those containing different S, C or P‐donor ligands have shown great cytotoxic potential against different tumours.[^5^, ^6^]

Alternatively, phosphorescent orthometallated iridium complexes have been investigated in many fields due to their outstanding optical properties.[^7^, ^8^, ^9^] High quantum yields, large Stokes shift and long emission lifetimes are some of the characteristics that make them very attractive for the development of light emitting devices, chemosensors or bioimaging probes.^[10]^ Their typical structure contains two bidentate cyclometallated ligands (C^N, e. g., 2‐phenylpyridine, ppy) and one bidentate diimine ligand (N^N, e. g., 2,2’‐bipyridine, bpy). Much effort is concentrated in the modification of those ligands to reach the desired optical probe for each particular application. In fact, there is a great variety of [Ir(N^C)_2_(N^N)]^+^ derivatives for tracking different organelles such as lysosomes,^[11]^ mitochondria,^[12]^ autophagic lysosomes,^[13]^ endoplasmic reticulum,^[14]^ or cytoplasmic localisation,^[15]^ among others. On the contrary, iridium complexes containing a diphosphine ligand instead of the diimine are far less studied, let alone in applications like bioimaging or therapy. Only very recently, Ir^III^ complexes of the type [Ir(C^N)_2_(P^P)]^+^ have revealed their tremendous prospect as theranostic agents, allowing the visualisation of their cellular biodistribution and, at the same time, displaying certain cytotoxicity towards different cancer cells.[^16^, ^17^]

**Figure 1:**
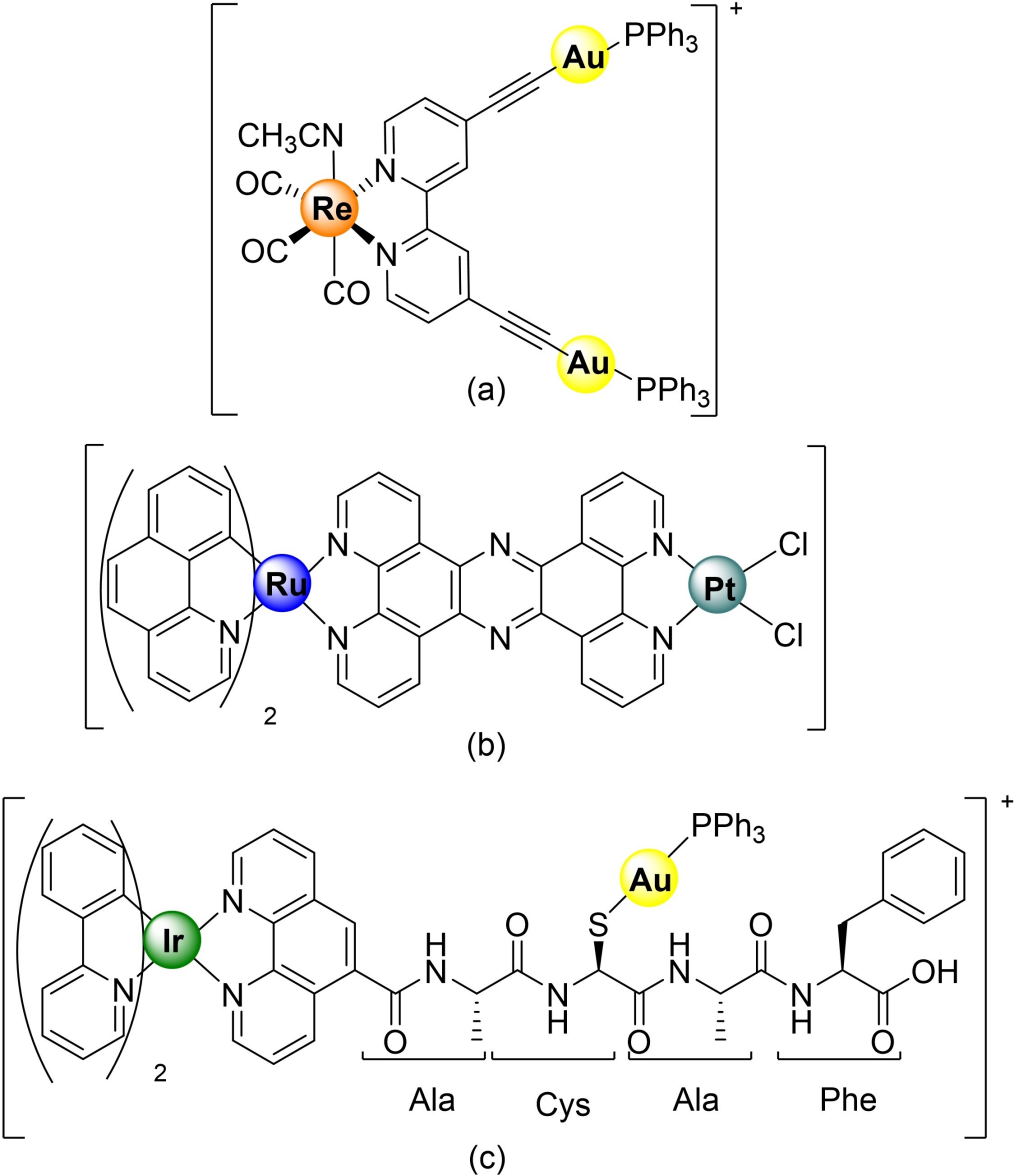
Recent examples of heterometallic theranostic agents. (a),^[19]^ (b),^[22]^ (c).^[23]^

An ingenious approach towards the development of a new generation of metallodrugs would be the combination of optically active and already cytotoxic metallic species with a different metallic fragment characterised for its therapeutic potential. A thorough selection of the coordination sphere of both metals will remain the key for the delivery of a selective theranostic probe targeting a specific organelle, as well as for a possible synergistic effect that would provide an exponential therapeutic effect on cancer cells. Up to date, heterometallodrugs in theranostics is still in their infancy.^[18]^ Only few examples have been published so far, and the majority describes the combination of either Re^I^ or Ru^II^ species as the emissive fragments with cytotoxic species based on Au^I^ or Pt^II^ (Figure 1).[^19^, ^20^, ^21^, ^22^] Regarding Ir^III^‐Au^I^ based hetero‐metallodrugs, a single example was recently reported, where a orthometallated Ir^III^ species of the type [Ir(C^N)_2_(N^N)]^+^ was functionalised with a peptide coupled to therapeutic Au^I^ fragment (Figure 1).^[23]^

Thus, in this work, combination of an emissive and already cytotoxic [Ir(C^N)_2_(P^P)]^+^ species with a biological relevant Au^I^ fragment is addressed to assess their antitumour potential as wells as their possible use as selective organelle theranostic probes. This research aims to open the door beyond the typical [Ir(C^N)_2_(P^P)]^+^ structures towards new Ir^III^−Au^I^ species containing diphosphino‐methanide ligands as linkers. These ligands allow an easy and robust coordination of the gold fragment while providing the possibility of having both cationic and neutral complexes. Studies of cytotoxicity, cell death mechanism, reactive oxygen species (ROS) production, mitochondrial membrane potential integrity and cell biodistribution in cancer cells (A549 cells) were performed for different gold ancillary ligands. Additionally, neonatal and adult vasculogenesis in presence of the novel metallodrugs was examined using endothelial colony forming cells (ECFCs) to assess their antiangiogenic potential and possible use as antimetastatic agents.

## Results and Discussion

### Synthesis and characterisation

The synthetic approach for developing organelle selective heterobimetallic Ir^III^−Au^I^ theranostic agents entails the use of bis(diphenylphosphino) methanide as linker between both metal fragments. Thus, the two phosphorous atoms bind the iridium metal centre in a bidentate chelate fashion, whereas the bioactive gold fragment would be directly coordinated to the methanide carbon upon deprotonation of the methylene.

**Scheme 1:**
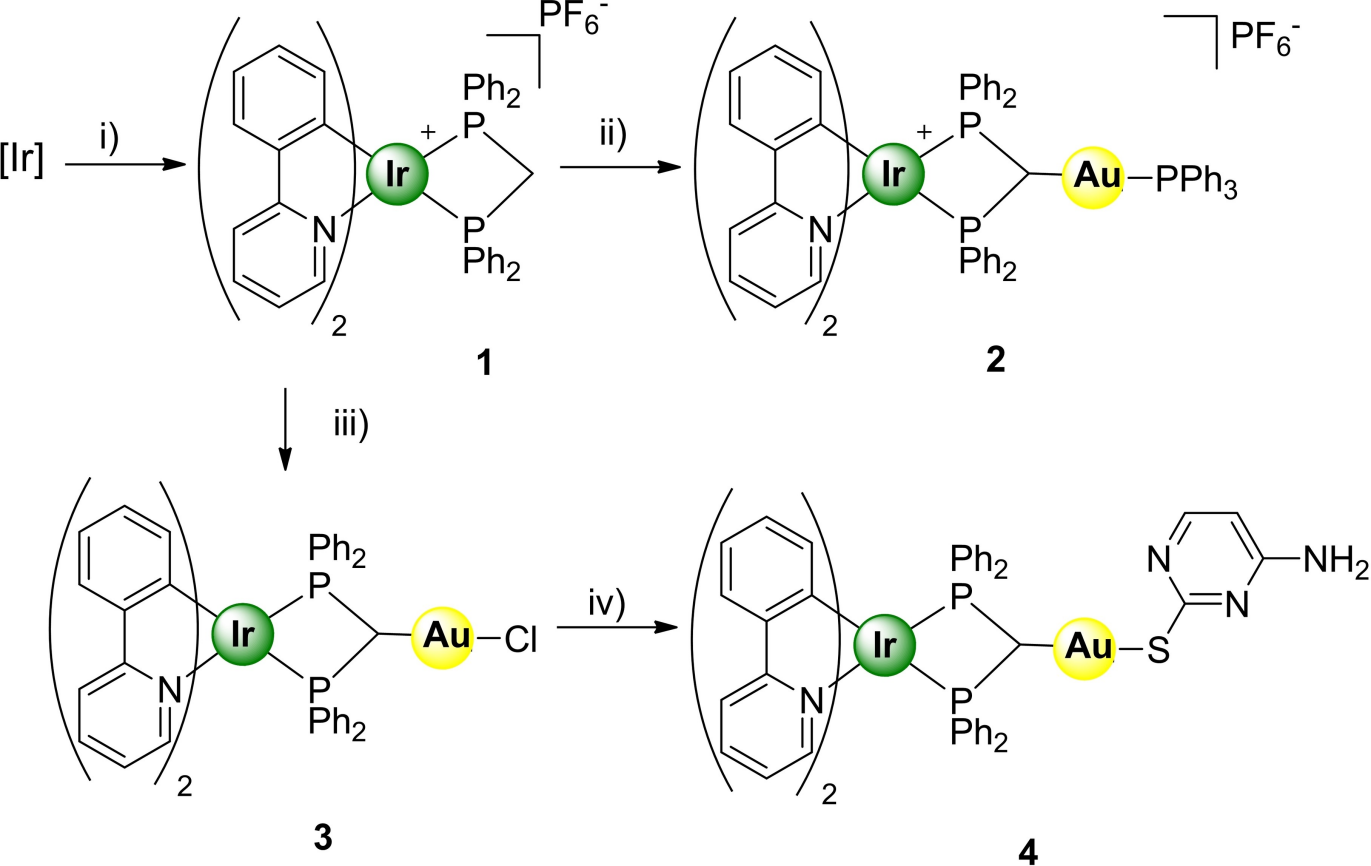
Synthesis and chemical structures of complexes **1**–**4**. i) [Ir]: [[Ir(ppy)_2_(μ‐Cl)]_2_], dppm, MeOH, reflux; ii) [Au(acac)PPh_3_], CH_2_Cl_2_, RT; iii) [AuCl(tht)], Cs_2_CO_3_, CH_2_Cl_2_, RT; iv) Cs_2_CO_3_, thiocytosine, CH_2_Cl_2_, RT.

Following slightly modified literature procedures, [Ir(ppy)_2_(μ‐Cl)]_2_[^24^, ^25^] and [Ir(ppy)_2_(dppm)]PF_6_ (**1**)^[16]^ were synthesised. Briefly, **1** was obtained in a single step by refluxing together dppm and [[Ir(ppy)_2_(μ‐Cl)]_2_] in dry and degassed methanol for 12 h. Then, subsequent additions of the different gold(I) substrates, either [Au(acac)PPh_3_], [AuCl(tht)] (tht=tetrahydrothiophene) in dichloromethane in presence of an intrinsic or extrinsic base, that is, acetylacetonate or Cs_2_CO_3,_ afforded complexes **2** and **3**, respectively (Scheme 1). Finally, complex **4** was obtained by a ligand exchange reaction, starting from complex **3** in CH_2_Cl_2_ and adding thiocytosine and Cs_2_CO_3_ as base, following an adapted published procedure.^[26]^ Thus, the chloride ligand was successfully substituted by the thiocytosine molecule coordinating the gold centre exclusively through the sulfur atom (Scheme 1).

**Figure 2:**
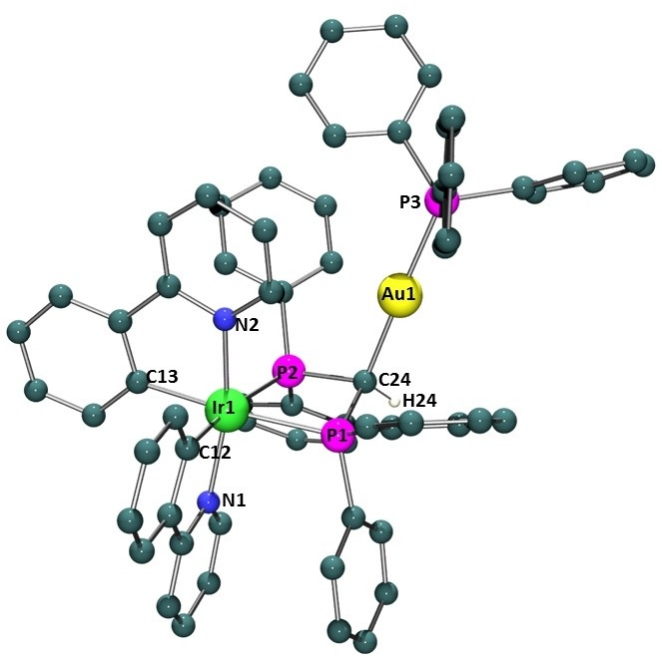
Pov‐ray representations of one of the molecules crystallised from complex **2**. Counterion, solvent molecules and hydrogens (except hydrogen 24) have been omitted for clarity. The most relevant bond lengths [Å] and angles [°]: Ir1‐C12: 2.063(7), Ir1‐C13: 2.059(7), Ir1‐N1: 2.079(6), Ir1‐N2: 2.068(5), Ir1‐P1: 2.388(5), Ir1‐P2: 2.421(2), Au1‐C24: 2.109(7), Au1‐P3: 2.274(1), C24‐Au1‐P3: 177.9(2), P2‐Ir1‐P1: 68.77(6), N2‐Ir1‐N1: 165.8(6), P1‐C24‐P1: 96.0(3).

The purity of the complexes was confirmed by ^1^H, ^13^C, and ^31^P NMR spectroscopy, IR spectroscopy when convenient and by HRMS analyses (Figures S1‐S16 in the Supporting Information). ^31^P NMR spectroscopy was particularly relevant for the characterisation of **2**. The singlet peak at −56 ppm, observed for the dppm ligand in complex **1**, shifted to lower field and reveals itself as doublet of doublets at −46 and −49 ppm in complex **2**, as a consequence of the inequivalence of the phosphorus atoms. In addition, a new peak appears at 38 ppm corroborating the presence of the PPh_3_ ligand (Figure S6). In the case of complex **3**, the characteristic υ(Au−Cl) absorption band observed at 335 cm^−1^ indicated the accomplishment of the gold coordination reaction.^[27]^ Moreover, in all cases ^1^H NMR spectroscopy showed that the methylene protons appearing at 5.92 ppm as a triplet in complex **1** shifted to either 5.42, 5.38 or 5.49 ppm because of the deprotonation and further coordination reaction of the respective gold fragment to afford **2**, **3** and **4** respectively. Additionally, suitable crystals of complex **2** for X‐ray diffraction were obtained by slow diffusion of diethyl ether into a dichloroethane solution of **2** (Figures 2 and S17). The molecule crystallised in the space group P‐1. The asymmetric unit is formed by two independent molecules of the Λ enantiomer and six molecules of dichloroethane from the crystallisation solvent. As expected for these types of iridium complexes, the coordinated ligands adopt an octahedral disposition around the metal centre. Specifically, the pyridyl nitrogen atoms from orthometallated ligand are coordinated to the Ir^III^ *trans* to each other with a N−Ir−N angle of 165.8(6)° (molecule 1) and 166.1(3)° (molecule 2), leaving phenyl carbons *trans* to the phosphorous atoms of the diphosphine ligand. Bond angles and distances from Ir^III^ coordination sphere of both independent molecules are similar to those already reported for a similar Ir^III^ biphosphine derivative,^[11]^ except for the P−Ir−P bite angle which is slightly smaller in the present case (ca. 68° vs. 70°). Possibly, coordination of the gold fragment to the methanide carbon is leading to a greater constriction around the Ir^III^ centre. In addition, Au^I^ is bonded to the already mentioned methanide carbon and the phosphorus atom from the triphenylphosphine ligand, displaying, as expected, a linear disposition. Once again, bond distances and angles from the Au^I^ fragment present values similar to those already reported.^[28]^ The most relevant crystallographic data together with selected bond distances and angles are collected in Table S1 and Figures 2 and S17.

### Optical properties

Photophysical properties of complexes **1**–**4** were analysed in DMSO solution at room temperature. The most significant data are collected in Table 1. The intense absorption bands observed below 300 nm were assigned to ligand centred spin‐allowed π→π* transitions within the ligands (Figure S18). Weaker transition at lower energies between 300 and 385 nm may be caused by intraligand ^3^π→π* and spin‐allowed ^1^MLCT transitions, with a tail extended over 400 nm, clearly observable for complex **4**, which is typically associated with a spin‐forbidden ^3^MLCT transition.^[29]^

**Table 1:** Photophysical data of **1**–**4** measured in aerated DMSO solution.

	UV/vis ×10^3^ (*ϵ* [dm^3^ mol^−1^ cm^−1^])	*λ*_em_ (*λ*_ex_) [nm]	*ζ* [ns]	*Φ*
1	265 (9.5), 304 (3.9), 384 (1.2)	526 (425)	181	1.6
2	265 (10.1), 305 (4.5), 382 (1.5)	528 (446)	160	5.1
3	260 (10.2), 300 (4.9), 374 (1.6)	527 (415)	234	2.7
4	263 (10.6), 303 (4.5), 385 (1.2)	529 (407)	184	1.2

**Figure 3:**
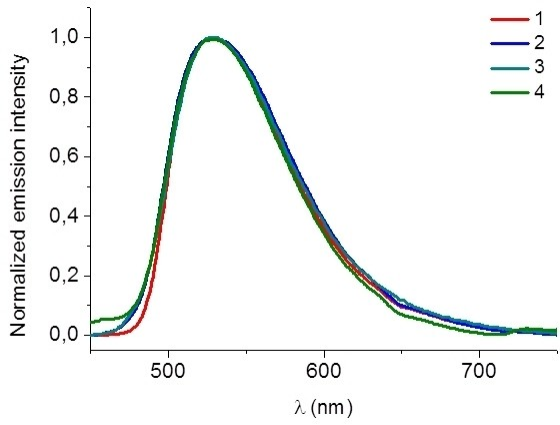
Emission spectra of complexes **1**–**4** measured in DSMO solution.

Regarding their emission properties, these complexes display a structureless emission centred ca. 527 nm (Figures 3 and S19). This emission is independent of the presence of the additional gold centre as well as the nature of its ancillary ligand. Generally, emission bands from charge transfer (CT) states are broad and featureless, while ligand‐centred (LC) states typically give highly structured emissions.^[30]^ Therefore, this emission is assigned mainly to a ^3^MLCT rather than ^3^LC transitions, which contrapose other iridium complexes containing diphosphine ligands.[^29^, ^31^, ^32^] The main difference in this particular case is the highly constricted iridium coordination sphere, due to the small chelate P−Ir−P angle, together with the nature of the anionic Ph_2_PCHPPh_2_^−^ bifunctional ligand. Such effects might be the reason for the prevalence of the ^3^MLCT character over that of ^3^LC. Similar effect was observed in highly constricted Ir complexes containing bent diphosphine chelators, leading to a structureless emission.^[33]^ Lifetime values ranged from 160 to 234 ns, corroborating the phosphorescent nature of the emission. Quantum yield measured in aerated DMSO revealed the higher emission efficiency for compound **2** (*Φ*=5.1) in comparison with their analogue complexes with quantum yields from 1.2 to 2.7 (Table 1). It seems clear that the gold ancillary ligand plays an important role on the modulation of the emission efficiency, the thiocytosine derivative being the one rendering the lowest quantum yield values.

### Stability studies in solution

Stability of the complexes was studied in neat DMSO and in a mixture of DMSO and phosphate‐buffered saline (PBS) solution (pH 7.4, final DMSO concentration ≤ 5 %) by UV/vis spectroscopy for a period of 48 h. No changes were observed on the specific absorption bands of the complexes, indicating their structural stability during the experiment (Figure S18).

### Cytotoxic activity and cell death mechanism in A549 cell line

In vitro bioactivity of complexes **1** to **4** as well as their dimer precursor was determined by MTT‐reduction assays in A549 lung carcinoma cells (Table 2). All complexes presented a significant cytotoxicity character, yielding IC_50_ values between 0.6 and 1.7 μM. On the contrary, neither their precursor, [Ir(ppy)_2_(μ‐Cl)]_2_ with IC_50_ values over 50 μM, nor the dppm,^[34]^ affected cell viability by themselves, pointing towards the combination of both entities to reveal the antiproliferative effect. Cells show an increased sensitivity to the heterobimetallic species bearing a phosphine or a thiocytosine group as gold ancillary ligand, complexes **2** and **4** respectively. The higher balance of the lipo‐ *vs* hydrophilicity of triphenylphosphine^[35]^ of complex **2**, or the bio‐friendly nature of the thiocytosine ancillary ligand[^36^, ^37^] in the case of complex **4** might promote an increase of the cell permeabilisation and thus display a greater antiproliferative activity.

**Table 2:** IC_50_ values of the dimer and complexes **1**–**4** incubated in A549 cells for 24 h.

Complex	A549 IC_50_ [μM]	Complex	A549 IC_50_ [μM]
dimer	>50	**3**	1.7±0.08
**1**	1.0±0.08	**4**	0.6±0.06
**2**	0.8±0.06	cisplatin^[38]^	20.8±2.1

Cell death mechanism was studied by flow cytometry for the monometallic Ir^III^ **1** and heterometallic Ir^III^‐Au^I^ **4** species in order to compare whether the presence of the gold fragment triggers a different cell death pathway. Previously to flow cytometry analysis, cells were visualised under an inverted microscope with the purpose of detecting significant changes in cell morphology and behaviour. Untreated cells were healthy and growing exponentially as expected; however, treatments with complexes **1** and **4** induced several perturbations in the cells (Figure S20). Both complexes promoted the generation of cytoplasmic vacuoles at 24 h, whose size increased with the dose and time, but little evidence of cell death was observed (data not shown). Nevertheless, even though cytoplasmic vacuolisation levels at 48 h after addition of the compounds were extremely high and the vacuoles reached such giant sizes that they extended throughout the entire cytoplasm. A priori, this observation seems to correlate with an increase in apoptotic and necrotic cell populations, which were more abundant at increasing doses (Figure S20).

**Figure 4:**
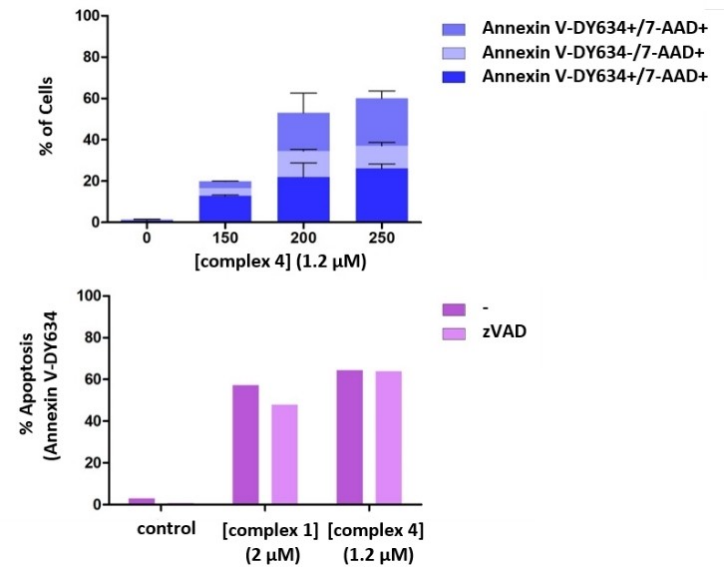
Cell death mechanism induced by complexes **1** and **4** in A549 cells. Top: cytotoxic effect of compound **4** evaluated with Annexin V‐DY634 and 7‐AAD staining. Bottom: percentage of apoptotic cells when pre‐incubated with 30 μM Z‐VAD‐fmk or not before adding the complex.

For flow cytometry analysis, phosphatidylserine exposure on cell surface as an “early apoptosis” marker and cell membrane damage as necrosis indicator were evaluated with Annexin V‐DY634 (apoptosis) and the DNA‐labelling dye 7‐AAD (necrosis) staining. As expected, both complexes induced a more potent cytotoxic effect at higher concentrations after 48 h of treatment compared to that observed after 24 h (data not shown). As shown in Figures 4 and S22 (top graphs), cells are distributed in three populations. Annexin V‐DY634‐only positive cells are indicative of an early apoptotic process, a 7‐AAD‐only labelled cell population represents exploded cells as a typical endpoint of necrotic cell death, and the doubly stained population implies that cells started being apoptotic and then eventually burst, a phenomenon known as “late apoptosis”. It is remarkable that late apoptosis state was easily reached at higher concentrations for both complexes. Therefore, these results confirm microscopy observations. As both compounds activate apoptotic pathway, we wanted to unveil whether caspases were implicated in the cell death process by using the pan‐caspase inhibitor Z‐VAD‐fmk. Interestingly, microscopy images and flow cytometry analysis laid bare the inability of Z‐VAD‐fmk to prevent cytoplasmic vacuole formation and apoptotic cell death induced by both compounds at 24 h (data not shown) and 48 h of treatment (Figure 4 (bottom) and S21), suggesting that complexes **1** and **4** trigger a caspase‐independent cell death mechanism.

Based on these data, we presume that a complementary cell death process named “paraptosis” might be occurring. Paraptosis is a newly described programmed cell‐death process characterised by the absence of apoptotic morphology such as nuclear fragmentation, apoptotic body formation and chromatin condensation, among other features. Moreover, paraptosis is an independent caspases mechanism that displays mitochondrial swelling and produces cytoplasmatic vacuolation mainly derived from endoplasmic reticulum.[^39^, ^40^] Despite several of these characteristics are in agreement with our observations, we identified cellular blebbing and apoptotic bodies in microscopy evaluation (black arrows in Figures S20 and S21) as well as annexin V labelling in flow cytometry analysis at 48 h of treatment but not at 24 h. Similarly, a study with an Alzheimer's disease cell model showed the presence of paraptotic cells at 24 h and with an increase in culture time, the presence of apoptotic cells after 48 h. In addition, a decrease in the expression levels of Bcl‐2 and Bax overexpression was observed at 48 and 72 h, suggesting that paraptosis finally unleashes mitochondrial apoptotic pathway.^[41]^ Therefore, in the present case, it can be concluded that complexes **1** and **4** mediate paraptosis with subsequent apoptotic cell death. These results may be extended to complexes **2** and **3** due to their similar chemical structure although analogous cytotoxicity tests are required.

### Cellular biodistribution in A549 cells

**Figure 5:**
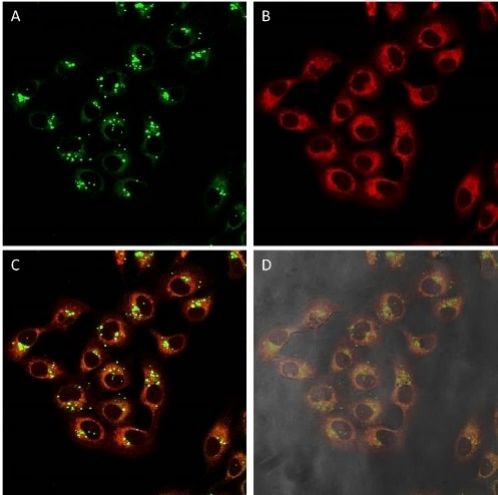
Fluorescence confocal microscopy images of A549 cells incubated with **1** (24 h) and stained with MTR. Image after irradiation at A) 473 and B) 598 nm. C) Superimposition of (A) and (B). D) Superimposition with phase contrast. Green: complex **1**, red: MTR; image width (↔): 210 μm.

In an attempt to elucidate the biodistribution of these complexes, fluorescence confocal microscopy assays were performed. Compounds were incubated in A549 cells for 24 hours. In addition to them, MitoTracker Red (MTR), a mitochondrial selective dye whose accumulation is dependent upon transmembrane potential, was added as internal standard. Superimposition of the images taken for the internal standard with those of the complexes revealed the cellular internalisation of the complexes. In all cases, the complexes enter the cells and get distributed within the cytoplasmic area, without permeating the nucleus. Although their emission pattern partly superimposes that of the MTR suggesting that they are partially localised in the mitochondria, they also accumulate in other areas of the cytoplasm. Thus, **1** presents a shocking pattern where the complex, apart from localising in the mitochondria, is accumulated in perfectly round shape spots organised close to the nucleus (Figure 5), this being their main localisation domain.

**Figure 6:**
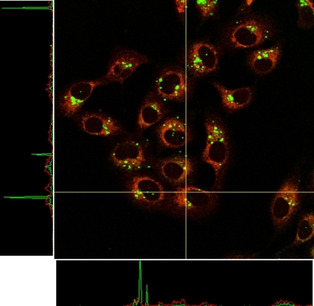
Cross section of intensity for the superimposition of compound **1** (green, irradiation at 473 nm) with MTR (red, irradiation at 598 nm) incubated in A549 cells. Image width (↔): 210 μm.

A closer look to the cross section intensity profiles of both fluorophores, MTR and **1** (Figure 6), seems to support the hypothesis that complex **1** is partially located in mitochondria. Nevertheless, the round spots observed with the compound **1** does not seem to correlate in first place with a mitochondrial localisation since the dotted pattern shown for MTR is thinner. Therefore, a plausible dual localization can be suggested. In fact similar complexes incubated with A549 cells have showed to deliver either mitochondrial^[16]^ or lysosomal^[17]^ localisation.

A similar pattern was displayed by complex **4**, although in this case the accumulation close to the nucleus is not as perfectly delimited in round shape spots (Figure S23). Despite that, and looking the cross section profiles of both dyes (Figure S24), there is again partial localisation in the mitochondria.

**Figure 7:**
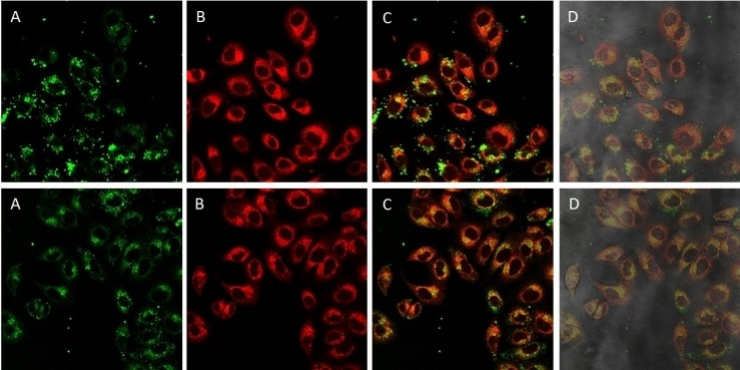
Fluorescence confocal microscopy images of A549 cells incubated with **2** (top) and **3** (bottom) for 24 h and stained with MTR. Images after irradiation at A) 473 and B) 598 nm. C) Superimposition of (A) and (B). D) Superimposition with phase contrast. Green: complexes **2** and **3**, red: MTR; image width (↔): 210 μm.

Regarding complexes **2** and **3**, they appear to be widely distributed throughout cells despite apparently staining mitochondria partly like their counterparts. In fact, their maxima emission is coming from spots randomly located in different parts of the cytoplasm, especially in the case of complex **2** (Figures 7 and S25).

In these colocalisation studies (Figures 5 and 7 and S23–25) it is important to notice that, the pattern arising from the MTR is completely different when comparing the images from the control cells and the cells treated with **1**–**4**. Thus, cells containing the complexes and MTR reveal a diffuse MTR distribution. On the contrary, control cells containing exclusively MTR, present a tubular mitochondrial distribution, synonymous of healthy mitochondria (Figure S26). This fact indicates that, effectively, the complexes have interfered with mitochondria integrity, limiting the MTR uptake.

**Figure 8:**
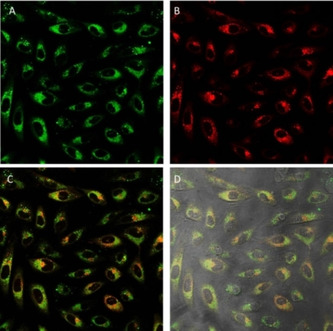
Fluorescence confocal microscopy images of A549 cells incubated with **1** (18 h) and stained with LTR. Image after irradiation at A) 473 and B) 598 nm. C) Superimposition of (A) and (B). D) Superimposition with phase contrast. Green: complex **1**, red: LTR; image width (↔): 210 μm.

To confirm the proposed dual localisation in lysosomes and mitochondria, a new colocalisation experiment was performed using LysoTracker Red‐DND‐99 (LTR) dye as internal standard. As expected, complexes **1** and **4** accumulate in the same organelles as LTR as well as other parts of the cytoplasm in a granulated fashion. Comparison with the previous MTR colocalisation assays suggests that both of them have at least dual localisation pattern, lysosomal and mitochondrial biodistribution (Figures 8 and S27).

**Figure 9:**
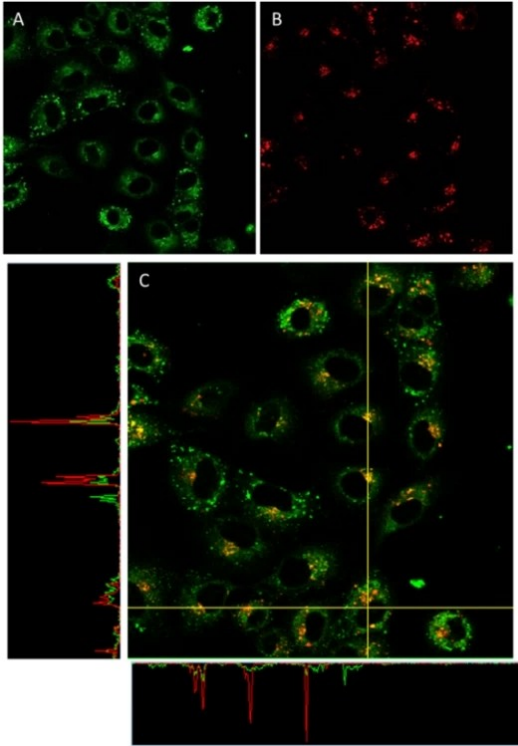
Fluorescence confocal microscopy images of A549 cells incubated with **3** (18 h) and stained with LTR. Image after irradiation at A) 473 and B) 598 nm. C) Superimposition of (A) and (B) and cross‐section of their intensities. Green: complex **3**, red: LTR; image width (↔): 210 μm.

Accordingly, cells incubated with complexes **2** and **3** also showed a similar biodistribution to these described for **1** and **4**, the localisation of **2** being slightly more aleatory in comparison with **1**, **3** and **4**. In any case, the cross section of the intensity of both complexes and LTR supports the partial lysosome accumulation (Figures 9 and S28).

**Figure 10:**
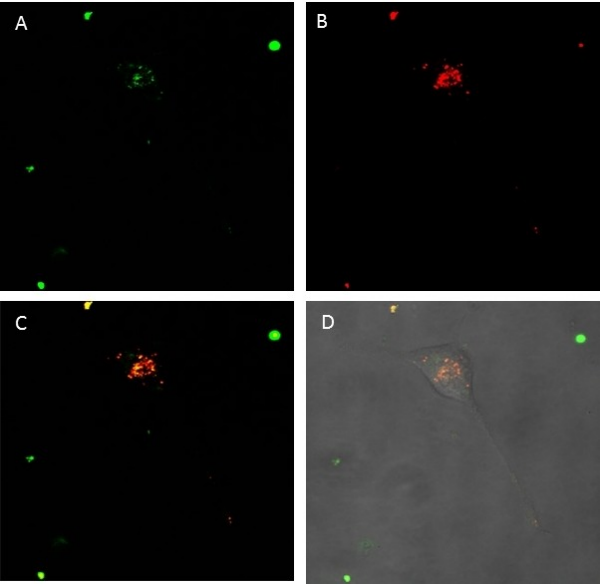
Fluorescence confocal microscopy images of A549 cells incubated with **4** (2 h) and stained with LTR. Image after irradiation at A) 473 and B) 598 nm. C) Superimposition of (A) and (B). D) Superimposition with phase contrast. Green: complex **4**, red: LTR; mage width (↔): 210 μm.

In view of the results, it can be assumed that all the complexes reveal at least a dual biodistribution in mitochondria and lysosomes. Migration process from the lysosomes to the mitochondria has been previously described for Ir^III^ complexes.^[42]^ Therefore, it cannot be ruled out that complexes **1**–**4** present a similar trafficking behaviour. In order to address the location of the complexes at first stages after entering the cells and to reduce to the minimum the emission from the subsequent mitochondrial trafficking process, a new colocalisation experiment with LTR was designed, this time reducing the incubation time up to 2 hours. Figure 10 again shows that incubation of complex **4** during short times delivers a partial emission pattern from the lysosomal area, indicating that complexes are localised in the lysosomes at initial stages after the uptake, probably due to an entry by endocytosis, and then the trafficking process to the mitochondria might take place. Unfortunately, there is not a certain explanation for the bright round spots observed for complexes **1** and **4**. It is clear that such peculiar accumulation is time dependent, as it is only observed at long incubation times (>18 h). Moreover, the fact that these spots weakly accumulates with MTR might suggest that mitochondria might be implicated after all. Additionally, both complexes are described to mediate paraptosis with subsequent apoptotic cell death. It is known that mitochondrial swelling and cytoplasmic vacuolation are paraptosis hallmarks.[^39^, ^40^] The fact that no emission was observed from the cytoplasmic vacuolization with an inverted microscope, suggest, once again, mitochondria and in particular swollen mitochondria, as source of the bright emissive round pattern.

### Generation of reactive oxygen species

**Figure 11:**
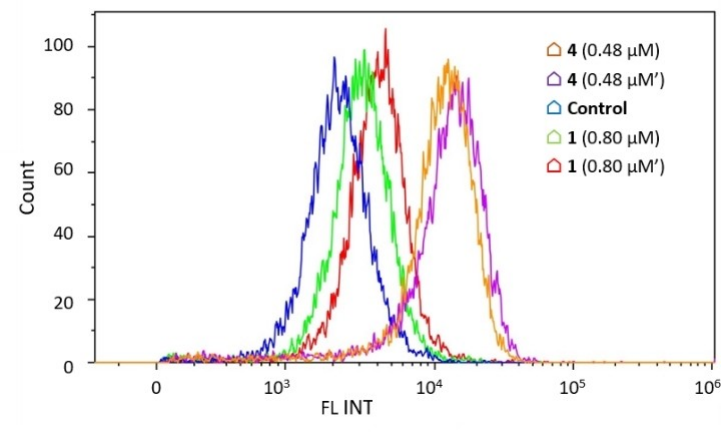
ROS production induced by **1** and **4** in A549 cells.

The generation of ROS was analysed by flow cytometry using a fluorogenic CellROX^TM^ assay kit. Complexes **1** and **4** were selected for the experiment to discriminate between the effects that might drive the presence of the gold fragment. A dose‐dependent increase in the generation of ROS in A549 cells after 24 hours of treatment was more evident for complex **1**. However, the levels of ROS found for complex **4** were much higher than in the case of complex **1** (Figure 11). Such increment might be in fact associated with the presence of the gold fragment as many reported gold species have shown to induce oxidative stress.^[43]^ Therefore, this experiment seems to indicate that the introduction of the gold fragment activates to a higher extent the mechanisms of ROS generation in A549 cells.

### Thioredoxin reductase inhibition

**Figure 12:**
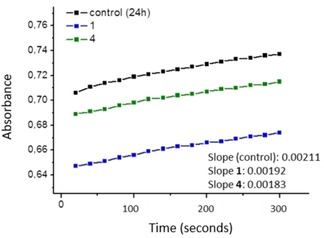
Inhibition of thioredoxin assay for complexes **1** and **4**. Representation of the evolution of the absorbance intensity of TNB over 5 min.

Gold complexes typically are known to inhibit the activity of the thioredoxin reductase TrxR.^[44]^ Therefore, complexes **1** and **4** were selected to study their possible interaction with this enzyme in order to elucidate, first if inhibition of the TrxR is taken place, and secondly, if the presence of the gold fragment is the driving force for the inhibition process. Both complexes were incubated with A549 cells for 24 hours and thereafter, the different total protein extracts were prepared and used for the determination of the TrxR activity. 5,5’‐Dithiobis(2‐nitrobenzoic acid) (DTNB) was added to the extracts in presence of NADPH and subsequent measurements of absorption intensity at 412 nm were performed by UV/vis spectroscopy. It is known that DTNB rapidly evolves to 2‐nitro‐5‐thiobenzoate anion (TNB) if the thioredoxin reductase has not been inhibited, affording a yellow solution that absorbs specifically at 412 nm.^[45]^ Figure 12 shows the value of the slopes for the fitted curve plot of the evolution TNB measured over a period of 5 min. Even though, both complexes display lower slope values in comparison to that of the control suggesting certain TrxR inhibition, it is complex **4**, as expected, the one affecting the activity of the TrxR to a greater extent.

### Mitochondrial transmembrane potential analysis

**Figure 13:**
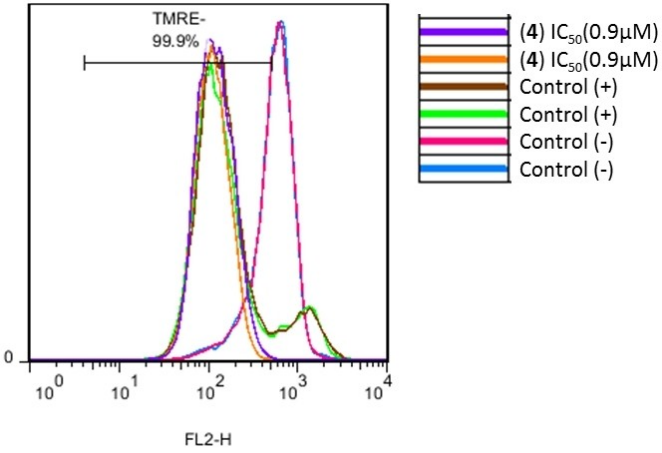
Disruption of mitochondrial transmembrane potential induced by **4**. Positive control: S63845.

As partial mitochondria localisation was observed for all the species and apoptotic cell death was detected, the integrity of the mitochondria was studied using the TMRE (tetramethylrhodamine ethyl ester) probe in order to analyse the mitochondrial transmembrane potential by flow cytometry. TMRE is a positively charged dye that accumulates in cells with active mitochondria as consequence of their net internal negative charge but several cell insults, including chemotherapeutic agents, lead to mitochondrial depolarisation and thus TMRE incorporation fails. A shift is seen in the pattern staining for complexes **1** (Figure S29) and **4** (Figure 13) in comparison with untreated cells and this overlaps with that seen when treating cells with S63845 (positive control), a selective inhibitor of the Mcl‐1 antiapoptotic protein involved in the regulation of the mitochondrial apoptotic pathway. Hence, these results point to the complete disruption of mitochondrial transmembrane potential driven by both species.

Mitochondrial transmembrane potential loss when treating cells with the complexes was also noticed in microscopy studies, as reflected by the poor MTR staining in Figures 5 and 7 and S23–25. Consequently, it becomes patently clear in both kind of assays that mitochondrial integrity is compromised in presence of the compounds. These results might correlate with cytotoxic effect induced by the complexes since the mitochondrial outer membrane permeabilisation is the point of no return in the mitochondrial apoptotic pathway and it comes with Annexin V positive labelling.[^46^, ^47^] Notwithstanding, we observed a total loss of the mitochondrial potential and a partial Annexin V staining after 48 h of exposure to the compounds, which denotes that other molecular mechanisms different from apoptosis are implicated and might be connected to paraptotic cell death already described.

### Biological properties in endothelial cells

Evaluation of drug cytotoxicity is a question of great importance regarding their intended target (i. e. tumour cells) but also other cell types that would be exposed to the drug throughout its dissemination by systemic circulation should be examined. Endothelial cells lining blood vessels may be susceptible to the potential cytotoxic effects of any blood transported compound. Several endothelial cell models are currently used to carry out this kind of analysis. Due to the diversity of endothelial phenotypes in the organism, no ideal cell system exists, and usually selection is made following convenience criteria. ECFCs (endothelial colony forming cells) are a subset of endothelial progenitor cells involved in angiogenesis and vasculogenesis both during neonatal and postnatal life. ECFCs may be efficiently obtained from both umbilical cord blood (ucb‐ECFCs) or adult peripheral blood (apb‐ECFCs) following careful cell culture protocols. Main differences between ucb‐ECFCs and apb‐ECFCs are their proliferative potential and the maturation degree, which can give rise to different physiological features. Therefore, they are primary cells that can reflect characteristics of the donor they come from, which is an interesting property to analyse variance of biological responses to drugs and to develop personalised medicine approaches.[^48^, ^49^, ^50^] It has been hypothesised that their functionality can be co‐opted by tumours in order to become vascularised, making them a target for therapies aimed to inhibit neovascularization as a strategy to control tumour growth.^[51]^ In fact, several reports have shown their possible involvement in the development of tumours by supporting tumour angiogenesis and metastasis.[^52^, ^53^, ^54^]

### Cell viability and cytotoxic effect in endothelial cells

Effects on the cell viability related to compounds **1**, **2** and **4** were analysed by crystal violet assays in ucb‐ECFCs and apb‐ECFCs. Both kinds of cells show a dose‐dependent sensitivity to the complexes after 24 h of treatment (Figure S30). IC_50_ values were on the range 1.1‐3.6 μM, ucb‐ECFCs being more sensitive than apb‐ECFCs to the compounds (Table 3), which could be associated to the increased proliferative activity of the ucb‐ECFCs. The ability of the different complexes to reduce cell viability does not seem to follow any specific trend regarding their mono or bimetallic character, complexes **1** (monometallic) and **4** (bimetallic) being more aggressive than complex **2** (bimetallic).

**Table 3:** IC_50_ values for complexes **1**, **2** and **4** incubated in ucb‐ECFCs and apb‐ECFCs for 24 h.

	IC_50_ [μM] (**1**)	IC_50_ [μM] (**2**)	IC_50_ [μM] (**4**)
ucb‐ECFCs	1.1640	2.6297	1.6599
apb‐ECFCs	2.7147	3.5224	2.3958

**Figure 14:**
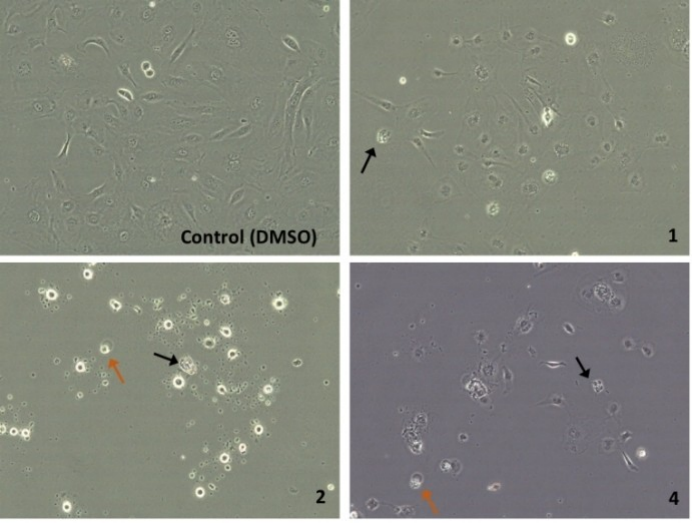
Phase‐contrast microscopy images of apb‐ECFCs incubated with **1**, **2** and **4** at 1 μM for 24 h.

In addition, cells were visualised under an inverted microscope to detect modifications in their morphology and behaviour as consequence of exposure to the compounds. Thus, ucb‐ECFCs and apb‐ECFCs were incubated with complexes **1**, **2** and **4** just below their IC_50_ during 24 h. Microscopy examination showed cell growth inhibition as well as apoptotic (black arrows) and necrotic cells (orange arrows) with all complexes (Figures 14 and S31). The effect of each compound was comparable for ucb‐ECFCs and apb‐ECFCs. Interestingly, complex **2** induced a different effect on cell morphology compared to compounds **1** and **4** but clear signs of cell death are still observed.

An apoptosis determination test was performed under acute conditions, that is, incubation for a short period of time (3 h) and high concentration of complexes (4 μM; Table 4). A different response was observed regarding the origin of the ECFCs. While the cytotoxic effect on ucb‐ECFCs was similar for all complexes (ca. 20 %), mortality levels of 50 % were achieved on apb‐ECFCs with complexes **1** and **4**. On the contrary, complex **2** appeared to be no toxic under these conditions.

**Table 4:** Determination of cell death for complexes **1**, **2** and **4** at 4 μM during 3 h.

	ECFCs	Viable	Early apoptotic	Late apoptotic	Necrotic
control	ucb	93.42 %	1.02 %	5.10 %	0.46 %
	apb	78.89 %	1.,79 %	10.29 %	0.05 %
**1**	ucb	73.10 %	1.12 %	21.81 %	3.96 %
	apb	30.75 %	8.30 %	51.93 %	9.02 %
**2**	ucb	76.82 %	0.93 %	17.24 %	5.01 %
	apb	82.25 %	9.22 %	8.40 %	0.14 %
**4**	ucb	72.29 %	2.14 %	22.32 %	3.26 %
	apb	27.88 %	10.35 %	51.06 %	10.71 %

These observations seem to be contradictory when compared to previous experiments carried out over 24 h time periods. However, these differences may be explained by differences in exposure times, the concentrations used for each compound and the parameters that each assay evaluates, since the crystal violet assay measures cell viability, the cytotoxic assay used determines two specific types of cell death (apoptosis and necrosis) and microscopy observations are limited to a narrow field of the entire well. In any case, these results point out that complex **2** acts differently from complexes **1** and **4** in both ucb‐ECFCs and apb‐ECFCs, and could involve other molecular mechanisms.

### In vitro angiogenic activity assays

**Figure 15:**
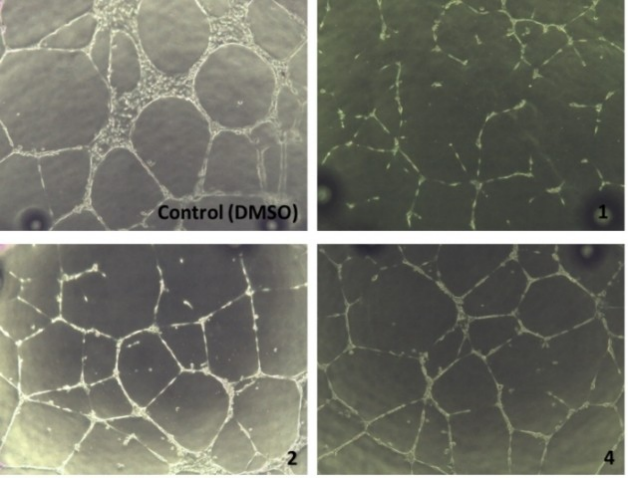
Phase‐contrast microscopy images of the in vitro angiogenic activity assays in ucb‐ECFCs cells incubated with **1**, **2** and **4** at 0.25 μM for 20 h.

In vitro tube formation assay is a highly informative test to probe angiogenic ability of endothelial cells.^[55]^ This observation could be the basis to consider their potential antiangiogenic activity and develop complementary assays in more physiologically relevant models. Tube activity formation of ECFCs on Cultrex hydrogel matrix was reduced although not completely impaired when compounds **1**, **2** and **4** were present in cell culture medium at a 0.25 μM concentration for 20 h. This effect happened regardless of the origin of the cells, but compounds **1** and **4**, once again, gave rise to a larger effect compared with **2** (Figures 15 and S32), thus suggesting their higher antiangiogenic character. The concentration of compounds used in these assays is an order of magnitude below the estimated IC_50_ values for ECFCs, so the inhibition of the angiogenic activity shown by the cells cannot be attributed to the cytotoxic effect of the compounds. The mechanisms involved in this effect deserve further research.

## Conclusions

In this work, three bioactive and emissive hetero‐bimetallic Ir^III^‐Au^I^ complexes (**2**‐**4**) containing a bis(diphenylphosphino) methanide ligand as linker between the metal fragments were developed. The complexes showed emissions dominated by their iridium fragment, with ^3^MLCT transitions, with no relevant shift of the emission maxima, but a small increase in the quantum yields in comparison with their parent monometallic Ir^III^ complex **1**. A549 cells showed high sensitivity to all complexes (0.6–1.7 μM) especially those containing the gold centre linked to a phosphine or a thiocytosine group. Interestingly, the introduction of the gold fragment prompted greater ROS generation and TrxR inhibition than the Ir^III^ analogue (**1**), but both types of compound managed to completely disrupt the mitochondrial transmembrane potential in A549 cells. Moreover, microscopy and cell death analysis performed in this cell line revealed massive cytoplasmic vacuolisation and apoptotic characteristics, but no caspases activation regardless of the presence of the gold fragment, thus opening the door to the involvement of other cell‐death processes like paraptosis. Biodistribution studies in A549 cells appear to show that complexes **1**–**4** exhibit at least a partial dual localisation in lysosomes and mitochondria. A time‐dependant trafficking process from the lysosomes to the mitochondria is suggested as complexes incubated for short times accumulate in lysosomes.

Additional cell‐viability assays in endothelial cells revealed that their sensitivity to the complexes does not follow any specific trend regarding their mono‐ or heterobimetallic character. All of them showed IC_50_ values ranging from 1.1 to 3.6 μM in both apb‐ECFC and ucb‐ECFCs, the latter being more sensitive to the complexes. Cytotoxicity studies pointed to apoptosis as the main cell‐death mechanism induced by the compounds in these endothelial cells. However, different responses were observed in the action of the compounds that could reflect the involvement of different molecular mechanisms depending on the cell type or compound used. Moreover, all of them displayed antiangiogenic properties in both endothelial cell types. Specifically, complexes **1** and **4** presented the highest inhibition of angiogenesis, revealing themselves as potential antimetastatic species. The different performances seen for these Ir^III^‐Au^I^ complexes in A549 and in apc‐ and ucb‐ECFCs evidences the high complexity involved in the design of novel theranostic agents, where not only the control of coordination spheres of the metallodrugs plays a crucial role, but also the biological material involved. These bimetallic complexes could be potential candidates for further studies as promising antitumour agents given their proven ability for disturbing mitochondria and raising ROS production, their potent cytotoxicity and antimetastatic activity. Finally, their activation of paraptosis in the initial stages of cell death opens the door to delivering alternative drugs tackling the massive problem of multidrug resistance, which is mainly to apoptotic phenotypes.

## Experimental Section

**Instrumentation**: Mass spectra were recorded on a BRUKER ESQUIRE 3000 PLUS, with the electrospray (ESI) technique. ^1^H and ^13^C{^1^H} NMR, including 2D experiments, were recorded at room temperature on a BRUKER AVANCE 400 spectrometer (^1^H, 400 MHz, ^13^C, 100.6 MHz, ^31^P, 162 MHz) with chemical shifts (δ, ppm) reported relative to the solvent peaks of the deuterated solvent.^[56]^ Steady‐state photoluminescence spectra were recorded with a Jobin‐Yvon‐Horiba fluorolog FL‐3‐11 spectrometer using band pathways of 3 nm for both excitation and emission. UV/vis spectra were recorded with 1 cm quartz cells on an Evolution 600 spectrophotometer. Quantum yields were measured using an absolute method provided by Hamamatsu Photonics Quantaurus‐QY C11347‐11. Specifically, each sample was measured using the excitation scanning mode in aerated DMSO solution at RT, after recording a reference sample (neat aerated DMSO at RT). Excitation of the samples was made from 370 to 470 nm in 10 nm intervals. The quantum yield value given is the one obtained at maxima intensity excitation, that is, **1** (425 nm), **2** (446 nm), **3** (415 nm), **4** (405 nm). The experiment was repeated three times to ensure reproducibility.

**Cell culture**: A549 (human lung carcinoma) cell line (from ATCC, USA) was routinely cultured in high glucose DMEM medium supplemented with 5 % foetal bovine serum (FBS), l‐glutamine and penicillin/streptomycin (hereafter, complete medium).

Endothelial colony forming cells (ECFC) were isolated from umbilical cord blood from healthy births but unsuitable for transplantation (ucb‐ECFCs), or from peripheral blood of adult healthy donors (apb‐ECFCs) following the method described by Ingram et al.^[57]^ with minor modifications. Briefly, blood was 1 : 2 diluted with Hanks solution (Sigma) and submitted to gradient density centrifugation using Histopaque 1077 (Sigma) to isolate PBMCs, which were subsequently washed with D‐PBS/BSA and seeded at a 150 000‐250 000 cells/cm^2^ density over type I rat collagen (Corning) coated cell culture dishes (TPP) on EGM®‐2 MV BulleKit®medium (Lonza) supplemented with 5 % FBS. Culture medium was changed daily during the first 7 days and every other day from then until the moment of first passage of the proliferating ECFCs to the dish, usually between 14 and 21 days after blood processing. Hidrocortisone was not used on ucb‐ECFCs. Before passaging cells were grown to 80 % confluence and passages 4–8 were used. All procedures were carried out under approval by the Ethical Committee for Clinical Research in Aragon (CEICA).

All cultures were maintained at 37 °C in a humidified atmosphere of 95 % air/5 % CO_2_.

**Cell viability assays** : The MTT‐reduction assay was used to analyse cell metabolic activity as an indicator for cell sensitivity to the complexes in A549 cell line. 10^5^ cells/mL were seeded in complete medium in flat‐bottom, 96‐well plates (100 μL/well) and allowed to attach for 24 h prior to addition of compounds. The complexes were dissolved in DMSO and added to cells in concentrations ranging from 0.5 to 50 μM in quadruplicate. Cells were incubated with our compounds for 24 h and then 10 μL of MTT (5 mg mL^−1^ in PBS) were added to each well and plates were incubated for 2 h at 37 °C. Finally, culture medium was removed and DMSO (100 μL/well) was added to dissolve the formazan crystals. The optical density was measured at 550 nm using a 96‐well multiscanner autoreader (ELISA) and IC_50_ was calculated. Each compound was analysed at least in three independent experiments.

For ECFCs cell viability determination, crystal violet assays were performed. These cells were seeded in 48‐well plates (Nunc) at a 50 000 cells/cm^2^ density and allowed to settle down and proliferate to 90 % confluence. Then, culture medium was changed by fresh medium containing vehicle (DMSO) or compounds in a range of concentrations between 0.125 and 8 μM and cells were treated during 24 h. DMSO concentration in the medium was below 0.5 %. After this incubation time, cells were fixed with glutaraldehyde 1 % and stained with crystal violet dye for 30 min, washed and plates were allowed to dry overnight. Estimation of cell density was achieved by solubilisation of cell‐retained crystal violet with 10 % acetic acid solution and absorbance measurement at 540 nm in a Synergy HD system (BioTek). Data were analysed with SAIC50 v1.5.10 (http://gauss.inf.um.es:8080/IC50/) using the four‐parameter logistic function to calculate IC_50_ values according to Sebaugh et al.^[58]^

**Cytotoxicity assays**: Apoptotic and necrotic cell death was determined by measuring phosphatidylserine exposure on cell surface and cell membrane permeabilisation, respectively, in A549 cells. 10^5^ cells/well were seeded in complete medium in flat‐bottom, 12‐well plates (1 mL/well) and left overnight to be attached to the bottom. Cells were treated for 48 h with compounds **1** and **4** at 1, 1.5, 2, 2.5 μM (**1**), 0.6, 0.9, 1.2, 1.5 μM (**4**) in duplicate. For apoptosis inhibition evaluation, cells were preincubated for 1 h with the pan‐caspase inhibitor Z‐VAD‐fmk (30 μM) (MedChemExpress, USA) prior to the addition of the compounds and it was renovated 24 h later. After treatments, cells were trypsinised, resuspended in 100 μL of a mixture of Annexin‐binding buffer (ABB; 140 mM NaCl, 2.5 mM CaCl_2_, 10 mM HEPES/NaOH pH 7.4), DY634‐conjugated Annexin V and 7‐amino‐actinomycin D (7‐AAD) and incubated at room temperature in the dark for 15 min. Finally, cells were diluted to 500 μl with ABB and a total of 10 000 cells were acquired on a FACSCalibur flow cytometer (BD Biosciences, USA). Cell death was analysed using CellQuest Pro (BD Biosciences), FlowJo 7.6.1 (Becton Dickinson (BD), USA) and GraphPad Prism 5 (GraphPad Software, USA) software.

For apoptosis assessment on ECFCs cells exposed to **1**, **2** and **4** complexes, an Annexin V apoptosis detection kit (Immunostep), with FITC‐conjugated Annexin V and propidium iodide (PI), was used. Cells were incubated during 3 h with **1**, **2** and **4** at 4 μM, gently detached from cell culture surface with Accutase (Corning) and resuspended in D‐PBS, and stained with FITC‐conjugated annexin V and PI according to manufacturer instructions. Data were acquired using a Gallios Flow Cytometer (Beckman Coulter), and Kaluza v2.1 (Beckman Coulter) software was used for data analysis.

**ROS production measurement**: 5×10^5^ A549 cells/well were seeded in complete medium in flat‐bottom, 6‐well plates (3 mL/well) and allowed to attach for 24 h prior to addition of the compound. Complexes **1** and **4** were dissolved in DMSO and added to cells up to concentration of 80 % (IC_50_) in duplicate. Cells were incubated with the complexes for 24 h and subsequently, they were trypsinised and the CellROX®Green Flow cytometry assay kit (C10492) was used to evaluate the generation of ROS in a Gallios Flow Cytometer (Beckman Coulter) using Kaluza v2.1 software for data analysis.

**Thioredoxin inhibition assay**: For determination of the thioredoxin reductase activity, A549 cells were incubated for 24 h with our compound at different concentrations near IC_50_ values. Cells were collected and washed with PBS and 150 μl buffer (1 % Triton X‐100, 150 mM NaCl, 50 mM Tris**⋅**HCl pH 7.6, 10 % *v*/*v* glycerol, 1 mM EDTA, 1 mM sodium orthovanadate, 10 mM sodium pyrophosphate, 10 μg mL^−1^ leupeptin, 10 mM NaF, 1 mM sodium methylsulfonium) for 30 min at 0 °C, and centrifuged at 200 *g* for 10 min at 4 °C. The protein was quantified using the BCA protein assay (Thermo Scientific) and 80 μg was added in each assay. Kinetic studies were performed in a buffer containing 0.2 M NaCl, H‐phosphate pH 7.4, 2 mM EDTA, 0.25 mM NADPH and 5 mM DTNB. The increase in the absorbance was measured at 412 nm for 5 min at 25 °C.

**Mitochondrial transmembrane potential analysis**: Integrity of the mitochondrial outer membrane was evaluated by measuring disruption of mitochondrial transmembrane potential. For this assay, 10^5^ A549 cells/mL were seeded in complete medium in flat‐bottom, 12‐well plates (1 mL/well) and allowed to attach for 24 h prior to addition of compounds. Cells were treated for 48 h with compound **4** at 150 μM in duplicate. Then, they were trypsinised, resuspended in 500 μl of a mixture of complete medium and 60 nM of the tetramethilrhodamine ethyl ester (TMRE) probe and incubated at 37 °C in the dark for 20 min. Finally, a total of 10 000 cells were acquired on a FACSCalibur flow cytometer (BD Biosciences) and data were analysed using the aforementioned software.

**Tube formation assay**: Endothelial cells (10 000 cell/well) suspended in cell culture medium containing each compound at 0.25 μM or vehicle (DMSO) were seeded over Cultrex® Reduced Growth Factor Basement Membrane Matrix (Trevigen) gelled (37 °C for 30 min) in wells of μ‐Slide Angiogenesis coverslips (ibidi). DMSO concentration in the medium was below 0.5 %. Cells were then incubated for a 20 h period with the treatments. Images were acquired with a Moticam 3 digital camera system (Motic) through a CK40 inverted microscope (Olympus) using a 10x objective.

**Fluorescence confocal microscopy**: 8×10^3^ A549 cells/well were seeded in complete medium in μ‐slide 8 well (ibiTreat) (300 μL/well) and left 24 h to be attached to the bottom. Then, 200 μL of culture medium was removed and 100 μL of a solution of the corresponding complexes were added to a final concentration of 2 μM. The complexes were incubated with the cells for 2 h, 18 h or 24 h depending on the specific experiment. Thereafter, MitoTracker Red (MTR) or LysoTracker Red‐DND‐99 (LTR) was added to a final concentration of 10 nM. They were incubated with the cells for 15 min (MitoTracker) or 30 min (LysoTracker) at room temperature. Eventually the medium was replaced with fresh medium without phenol red. Images were collected in a sequential mode in a FluoView FV10i (Olympus) confocal microscope with a 60 oil immersion lens, a line average of 4, and a format of 1024x1024 pixels using excitation wavelength of either 473 or 559 nm. The confocal pinhole was 1 Airy unit. Images were analysed with FV10‐ASW 3.1. Viewer software.

**Starting materials**: The starting material [Ir(ppy)_2_(μ‐Cl)]_2_,^[25]^ [AuCl(tht)][^59^, ^60^] and [Au(acac)PPh_3_]^[61]^ was prepared according to published procedures respectively and their experimental data agrees with that reported somewhere else. All other reagents were commercially available. Solvents were used with purification and drying, and all manipulations were performed in argon atmosphere.

### General synthetic procedures

*Synthetic procedure of complex **1***: The synthesis of 1 was accomplished by the treatment of two equivalents of ligand dppm (bis(diphenhylphophino) methane) (35.9 mg, 0.094 mmol) with one equivalent of [Ir(ppy)_2_(μ‐Cl)]_2_ (50 mg, 0.047 mmol) in methanol, under a reflux for 12 h. Once the reaction was completed, the solvent was evaporated under vacuum and the slurry was dissolved in the minimum amount of dichloromethane. Finally, hexane was added to obtain a pale yellow solid by precipitation of complex **1**. (82.7 mg, 0.08 mmol, 85 %). ^1^H NMR (400 MHz, CD_2_Cl_2_): *δ*=8.15 (d, *J*=5.4 Hz, 1H), 7.86 (dt, *J*=10.8, 4.8 Hz, 2H), 7.79–7.68 (m, 2H), 7.64–7.55 (m, 1H), 7.55–7.46 (m, 3H), 7.16–6.90 (m, 7H), 6.49–6.38 (m, 2H), 5.92 (t, *J*=9.8 Hz, 1H) ppm. ^13^C NMR (101 MHz, CD_2_Cl_2_): *δ*=156.6, 155.7, 153.6, 143.4, 138.4, 132.5, 132.3, 132.3, 131.6, 131.1, 130.6, 130.4, 130.3, 129.8, 129.5, 129.4, 129.3, 128.1, 125.5, 124.1, 120.9, 34.9 ppm. ^31^P NMR (162 MHz, CD_2_Cl_2_): *δ*=−56.11 ppm. ^19^F NMR (376 MHz, CD_2_Cl_2_): *δ*=−72.05, −73.94 ppm. HRMS (ESI): *m*/*z* calcd for C_47_H_38_IrN_2_P_2_: 885.1889; found: 885.2139.

*Synthetic procedure of complex **2***: A mixture of complex **1** (10 mg, 0.01 mmol) and [Au(acac)PPh_3_] (5.42 mg, 0.01 mmol) in dry dichloromethane (5 mL) was stirred for 8 h under argon atmosphere. The resultant solid was filtered and dried, affording complex **2** (13.56 mg, 0.009 mmol, 91 %). ^1^H NMR (400 MHz, CD_2_Cl_2_): *δ*=9.54 (d, *J*=5.6 Hz, 1H), 7.97 (d, *J*=7.6 Hz, 1H), 7.83–7.71 (m, 5H), 7.63 (dd, *J*=11.8, 7.6 Hz, 2H), 7.55–7.31 (m, 16H), 7.23–7.05 (m, 6H), 7.01–6.79 (m, 15H), 6.64 (td, *J*=7.9, 2.3 Hz, 2H), 6.46–6.32 (m, 1H), 6.28–6.19 (m, 1H), 6.03–5.97 (m, 1H), 5.52–5.37 (m, 1H) ppm. ^13^C NMR (101 MHz, CD_2_Cl_2_): *δ*=143.4, 142.6, 137.8, 137.6, 136.8, 136.6, 133.7, 133.6, 132.6, 132.5, 131.9, 131.6, 130.8, 130.7, 130.6, 130.4, 130.3, 130.1, 129.8, 129.7, 129.4, 129.3, 129.2, 128.6, 128.5, 128.1, 128.0, 127.9, 127.8, 124.6, 124.2, 123.2, 123.1, 122.7, 121.9, 119.9, 119.1, 34.3 ppm. ^31^P NMR (162 MHz, CD_2_Cl_2_): *δ*=38.62 (t, *J=* 9.5 Hz), −45.79 (dd, *J=* 37.8, 9.4 Hz), −48.73 (dd, *J=* 37.7, 9.7 Hz) ppm. HRMS (ESI): *m*/*z* calcd for C_65_H_52_AuIrN_2_P_3_: 1343.2615; found: 1343.2638.

*Synthetic procedure of complex **3***: A 50 mg (0.05 mmol) sample of **1**, 15.51 mg (0.05 mmol) of [AuCl(tht)], 15 mg of Cs_2_CO_3_ and 10 mL of dichloromethane were placed in a 50 mL flask. The mixture was stirred for 6 h at room temperature. The reaction was monitored by TLC (dichloromethane/ hexane 9 : 1). After the reaction was completed, diethyl ether was added to the mixture affording the precipitation of a yellow solid, complex **3**, which was filtered and dried. (38.79 mg, 0.034 mmol, 70 %). ^1^H NMR (400 MHz, CD_2_Cl_2_): *δ*=8.15 (d, *J*=5.6 Hz, 1H), 7.77–7.63 (m, 4H), 7.55 (dt, *J*=16.6, 8.0 Hz, 4H), 7.10 (d, *J*=7.5 Hz, 2H), 7.05–6.94 (m, 3H), 6.79 (d, *J*=10.2 Hz, 2H), 6.52–6.45 (m, 1H), 6.45–6.40 (m, 1H), 5.38 (d, *J*=7.3 Hz, 1H) ppm.^13^C NMR (101 MHz, CD_2_Cl_2_): *δ*=153.0, 147.2, 141.9, 140.5, 131.9, 131.7, 131.1, 130.5, 130.3, 130.0, 129.8, 129.3, 129.0, 128.8, 128.7, 124.9, 123.5, 123.4, 122.9, 122.3, 120.3, 118.7, 34.4 ppm. ^31^P NMR (162 MHz, CD_2_Cl_2_): *δ*=−56.85 ppm. IR: *ν*(Au−Cl): 335 cm^−1^).

*Synthetic procedure of complex **4***: 25 mg (0.02 mmol) of complex **3** were dissolved in 10 mL of dichloromethane and 5 equivalents of Cs_2_CO_3_ were added. The mixture was stirred for 15 min at room temperature. Then, thiocytosine (2.60 mg, 0.02 mmol) was added and the reaction mixture was stirred for 20 h. Once the reaction was completed, the solvent was evaporated to a minimum volume and the addition of diethyl ether afforded the precipitation of a pale yellow solid, which was filtered, washed with hexane and dried. (22.37 mg, 0.018 mmol, 92 %). ^1^H NMR (400 MHz, CD_2_Cl_2_): *δ*=8.15 (d, *J*=5.3 Hz, 1H), 7.78–7.63 (m, 5H), 7.55 (m, 5H), 7.11 (t, *J*=7.5 Hz, 2H), 6.99 (dt, *J*=13.6, 7.4 Hz, 3H), 6.79 (t, *J*=9.1 Hz, 2H), 6.48 (ddd, *J*=7.5, 6.0, 1.5 Hz, 1H), 6.43 (dd, *J*=7.5, 2.9 Hz, 1H), 5.39 (t, *J*=9.4 Hz, 1H). ^13^C NMR (101 MHz, CD_2_Cl_2_): *δ*=153.6, 148.3, 147.8, 141.3, 140.6, 138.9, 137.0, 134.6, 133.5, 133.1, 132.5, 132.3, 131.8, 130.9, 130.4, 129.8, 129.5, 127.3, 126.3, 125.5, 124.1, 123.8, 121.4, 120.9, 119.3, 117.99, 35.0. ^31^P NMR (162 MHz, CD_2_Cl_2_): *δ*=−56.71 ppm. HRMS (ESI): *m*/*z* calcd for C_47_H_41_AuIrN_2_P_2_S: 1117.1403; found: 1117.1760.

**Crystal structure analysis**: Crystals were mounted in inert oil glass fibres and transferred to the cold gas stream of an SMART APEX Duo diffractometer equipped with a low‐temperature attachment. Data were collected using monochromated Mo Kα radiation (λ=0.71073 Å). Scan type ω. Absorption corrections based on multiple scans were applied with the program SADABS.^[62]^ The structures were solved by direct methods and refined on F^2^ using the program SHELXL‐2016.^[63]^ All non‐hydrogen atoms were refined anisotropically. Hydrogen atoms were included in calculated positions and refined using a riding model. Refinements were carried out by full‐matrix least‐squares on F^2^ for all data. Deposition number 2052537 contains the supplementary crystallographic data for this paper. These data are provided free of charge by the joint Cambridge Crystallographic Data Centre and Fachinformationszentrum Karlsruhe Access Structures service.

## Conflict of interest

The authors declare no conflict of interest.

## Supporting information

As a service to our authors and readers, this journal provides supporting information supplied by the authors. Such materials are peer reviewed and may be re‐organized for online delivery, but are not copy‐edited or typeset. Technical support issues arising from supporting information (other than missing files) should be addressed to the authors.

Supplementary
