# Supplementary material for: Multifunctional Heterometallic IrIII−AuI Probes as Promising Anticancer and Antiangiogenic Agents
Source: Chemistry. 2021 May 29;27(38):9885–97. doi: 10.1002/chem.202100707 (PMC8361937; doi:10.1002/chem.202100707)
Supplement: Supplementary file 1 — Supplementary [file CHEM-27-9885-s001.pdf]

Chemistry–A European Journal

Supporting Information

Multifunctional Heterometallic Ir^{III}–Au^{I} Probes as Promising Anticancer and Antiangiogenic Agents

Marta Redrado, Andrea Benedi, Isabel Marzo, Angel L. García-Otín,* Vanesa Fernández-Moreira,* and M. Concepción Gimeno*

Electronic Supporting Information (ESI)

Table of contents

	**Page**
Figure S1-S13: NMR spectra of compounds **1-4**	**3**
Figure S14-S16: Mass spectra of compounds **1**, **2** and **4**	**11**
Table S1: Crystallographic data of complex **2**	**12**
Figure S17: Pov-ray representations of molecule Ir(2) crystallised from complex **2**	
Figure S18. Absorption spectra and stability studies of complexes **1-4**	**13**
Figure S19. Emission-excitation spectra of complexes **1-4**	**13**
Figure S20 Phase contrast microscopy images of A549 cells treated with 1 and 4 at different concentrations for 48 h.	**14**
Figure S21. Phase contrast microscopy images of A549 cells preincubated with Z-VAD (50 μM) and treated with 1 (2 μM) and 4 (1.5 μM) for 48 h.	**14**
Figure S22. Cell death assay using Annexin V-DY634, 7-AAD	**15**
Figure S23. Fluorescence confocal microscopy images of A549 cells incubated with **4** (24 h) and stained with MTR	**15**
Figure S24. Cross section of intensity for the superimposition image of complex **4** with MTR in A549 cells	**16**
Figure S25. Cross section of intensity for the superimposition image of complex **2** with MTR in A549 cells	**16**
Figure S26. Control cells incubated only with MTR	**17**
Figure S27. Fluorescence confocal microscopy images of A549 cells incubated with **4** (18 h) and stained with LTR	**17**
Figure S28. Fluorescence confocal microscopy images of A549 cells incubated with **2** (18 h) and stained with LTR	**18**

Figure S29. Study of the mitochondrial transmembrane potential integrity in A549 cells with complex **1** **18**
Figure S30. Cell viability of ucb-ECFCs and apb-ECFCs in presence of complexes **1**, **2** and **4** **19**
Figure S31. Phase contrast microscopy images of ucb-ECFCs incubated with **1**, **2** and **4** at 1 µM for 24 h **20**
Figure S32. Phase contrast microscopy images of the *in vitro* antiangiogenic activity assays in apb-ECFCs cells with **1**, **2** and **4** **20**

NMR spectra of compounds 1-4

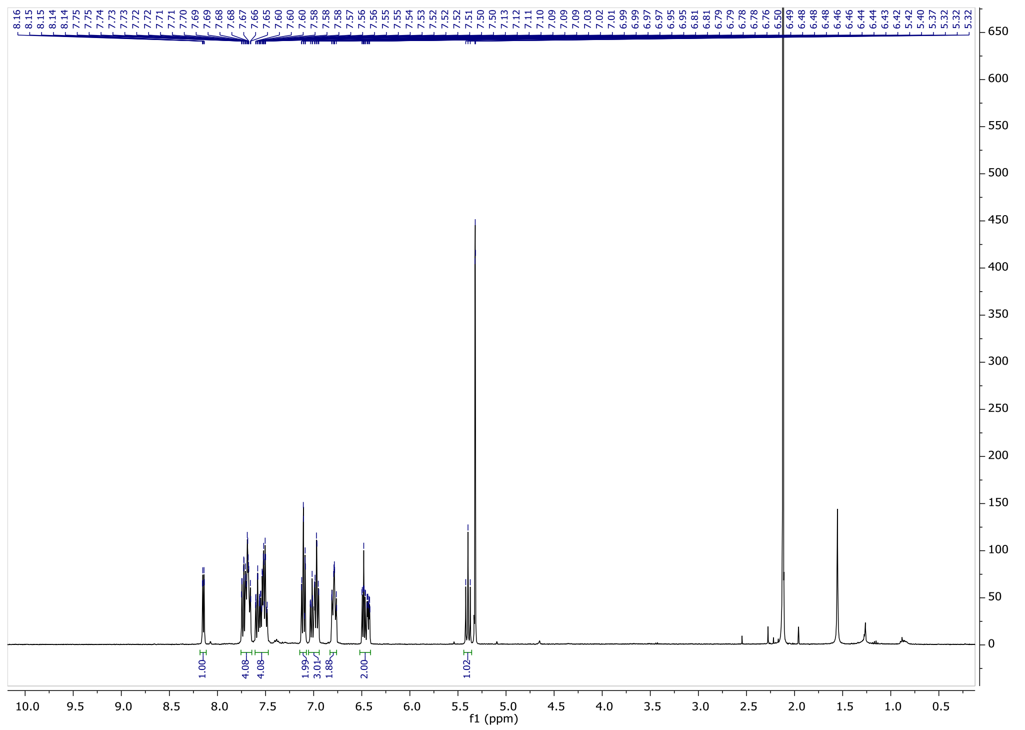

Figure S1. ^{1}H-NMR spectrum of complex **1**.

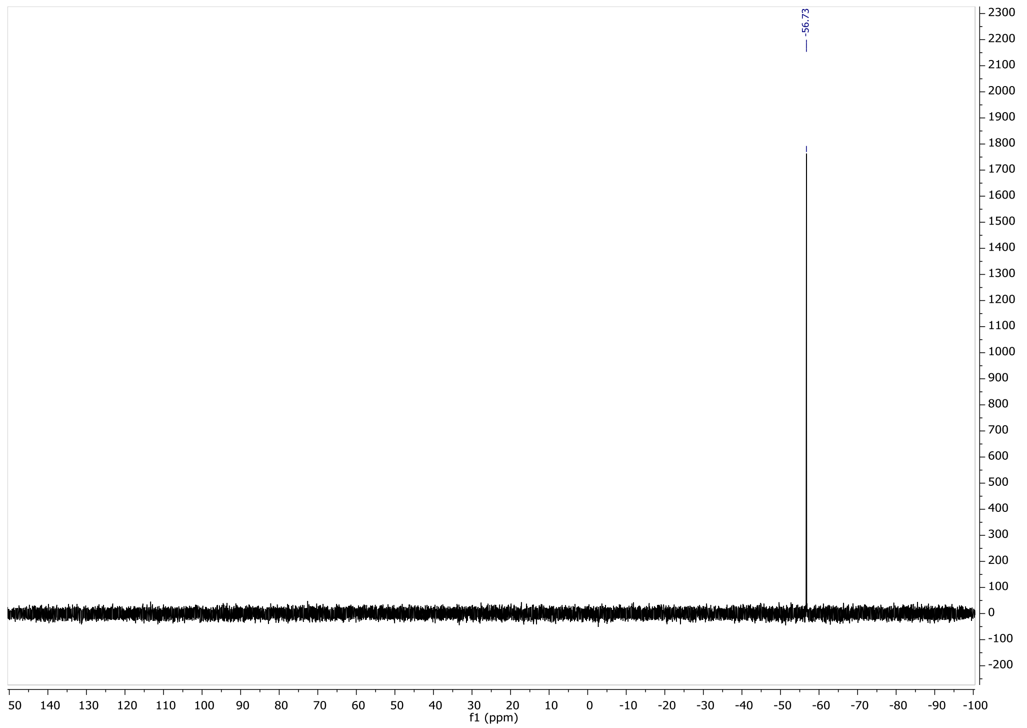

Figure S2. ^{31}P-NMR spectrum of complex **1**.

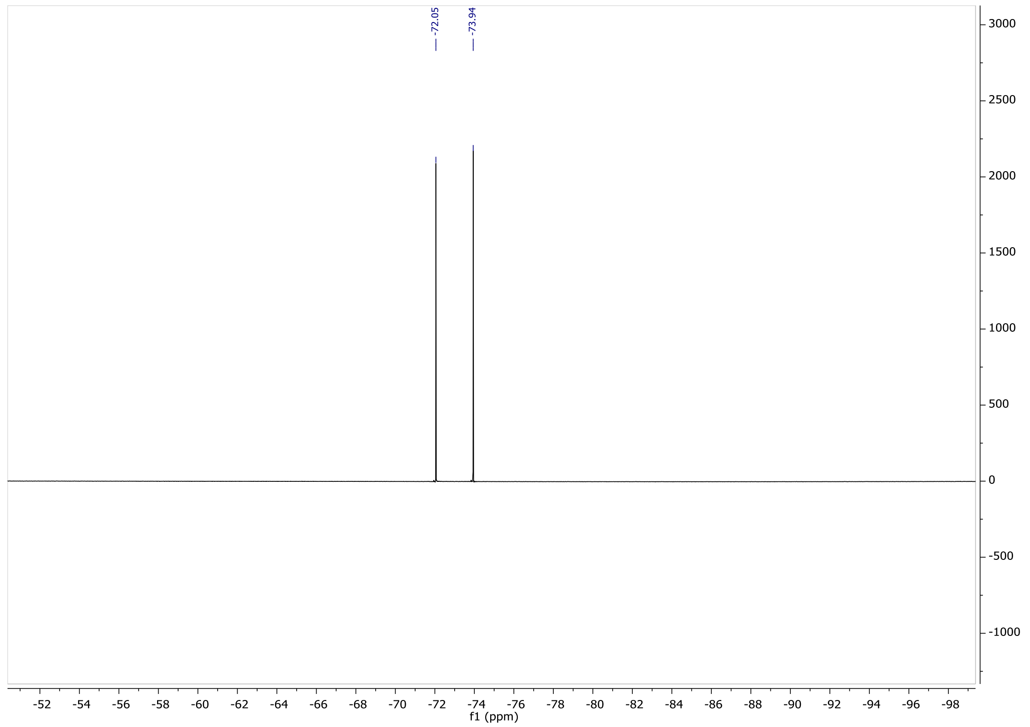

Figure S3. ^{19}F-NMR spectrum of complex **1**.

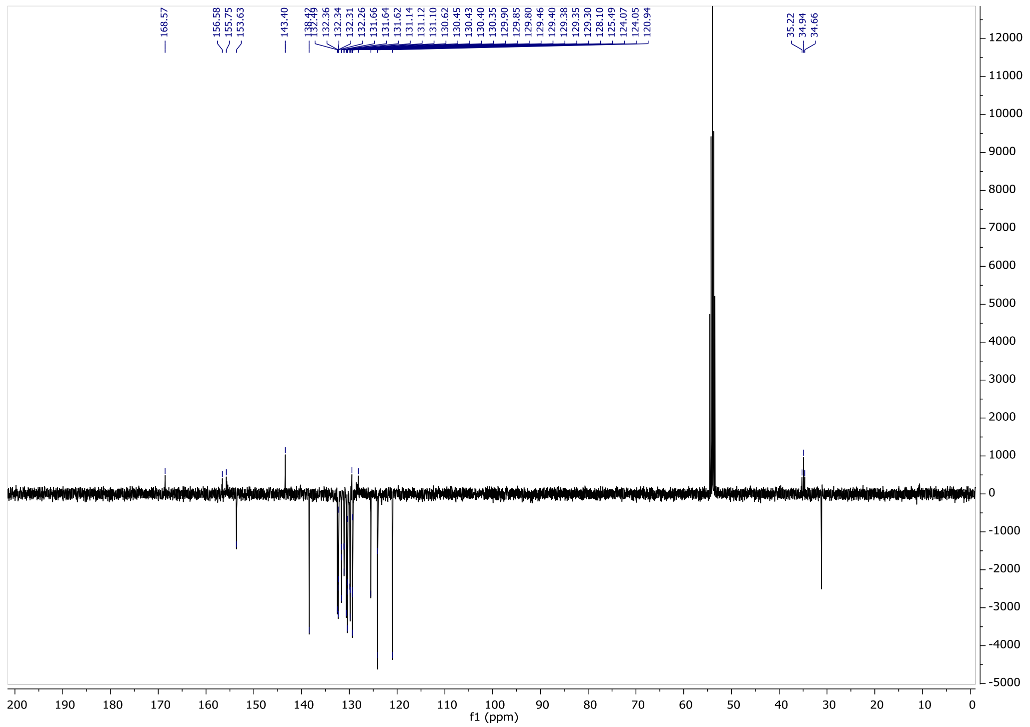

Figure S4. APT-NMR spectrum of complex **1**.

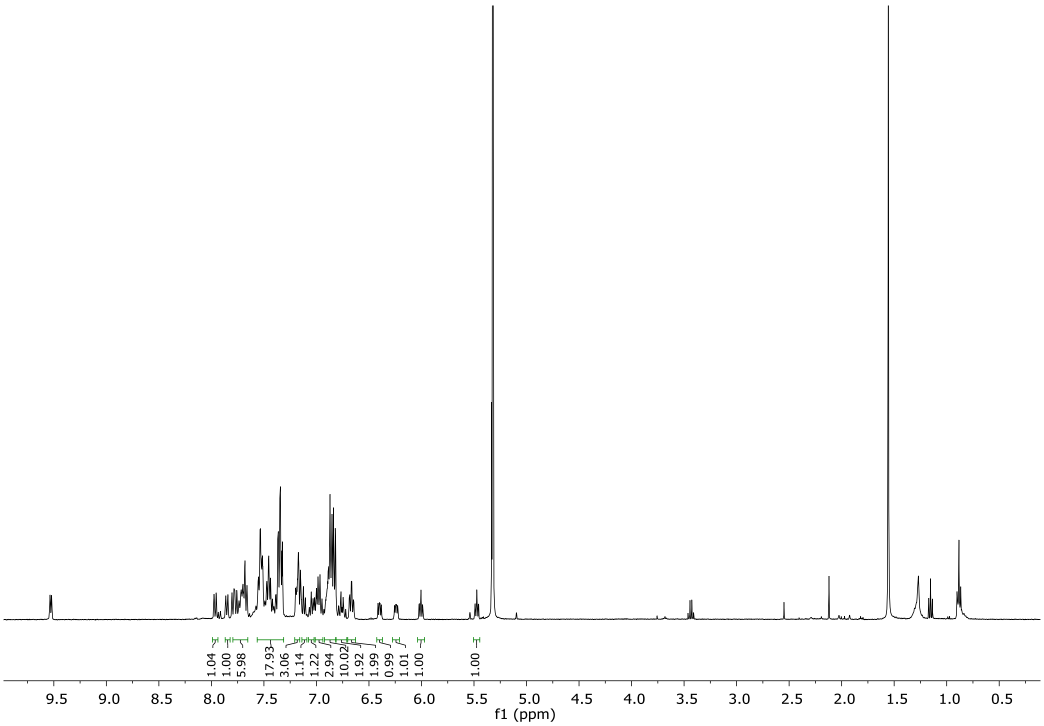

Figure S5. ^{1}H-NMR spectrum of complex **2**.

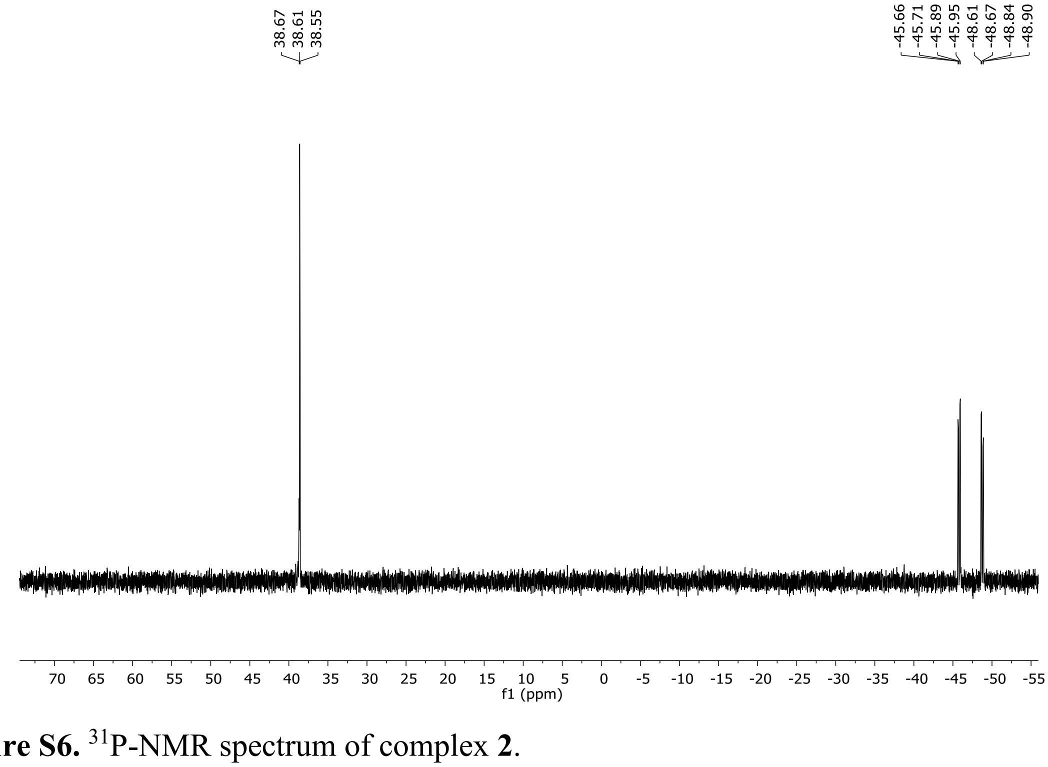

Figure S6. ^{31}P-NMR spectrum of complex **2**.

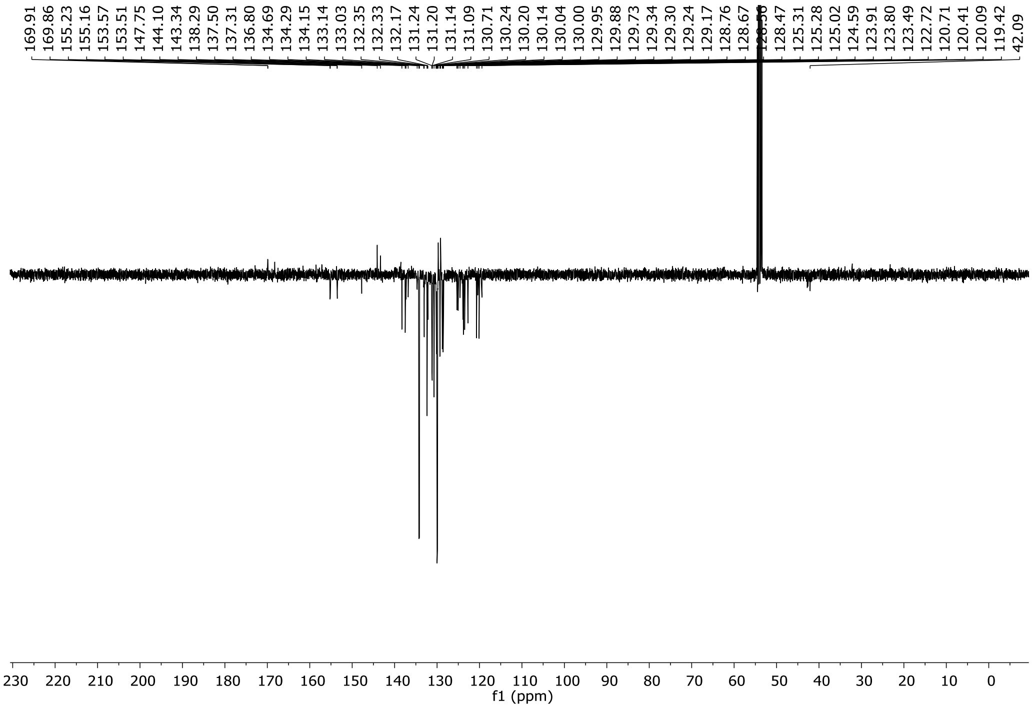

Figure S7. APT-NMR spectrum of complex **2**.

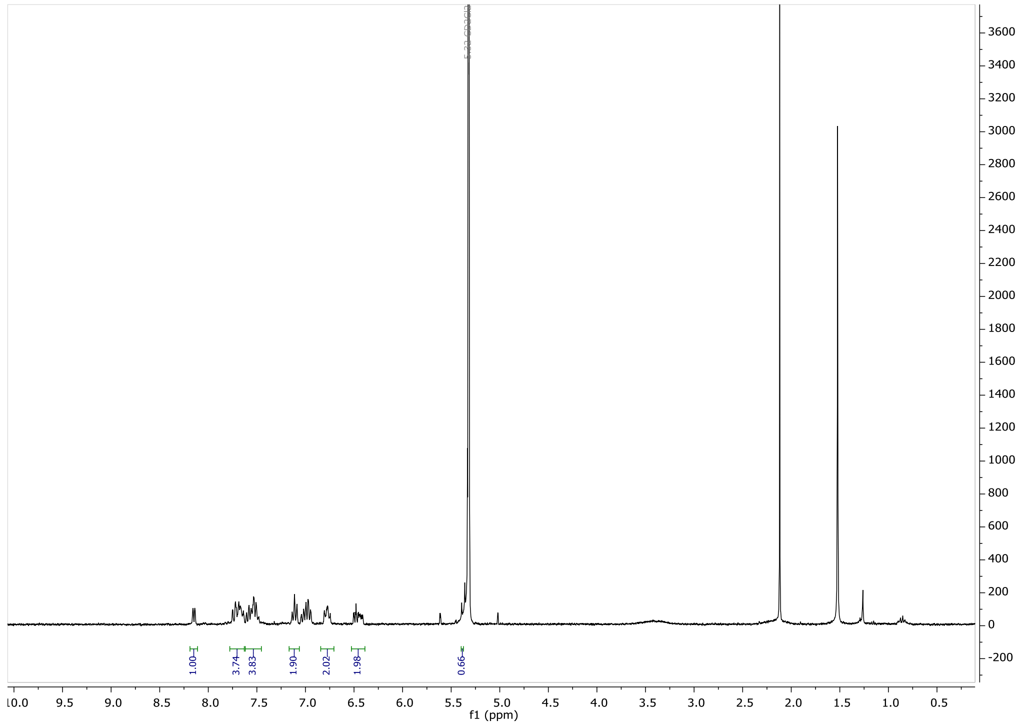

Figure S8. ^{1}H-NMR spectrum of complex **3**.

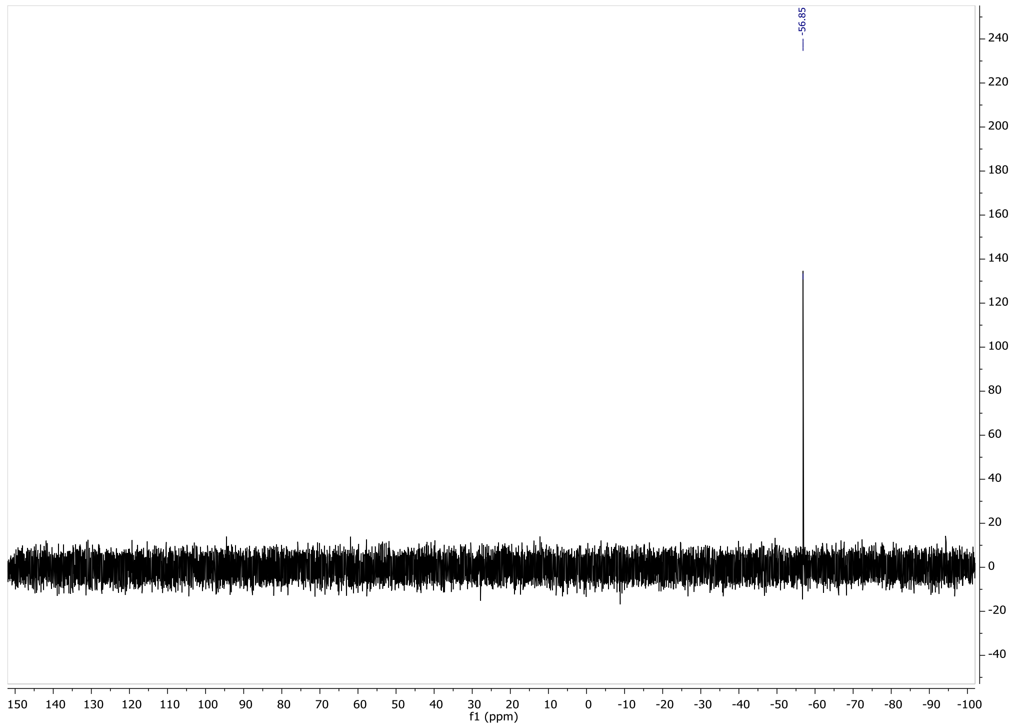

Figure S9. ^{31}P-NMR spectrum of complex **3**.

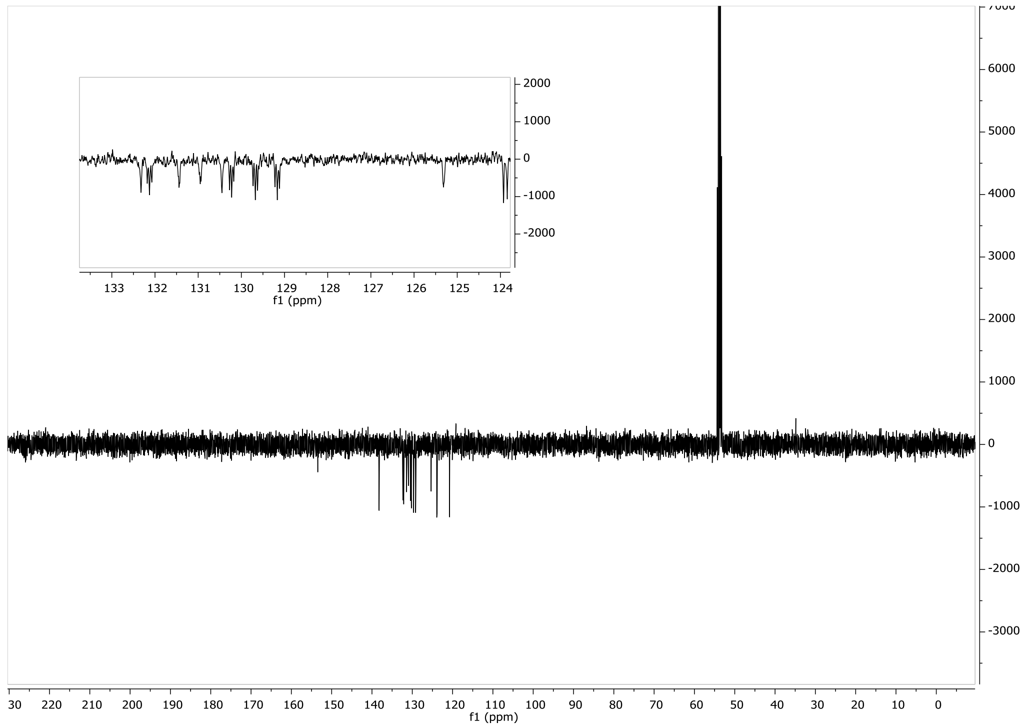

Figure S10 APT-NMR spectrum of complex **3**.

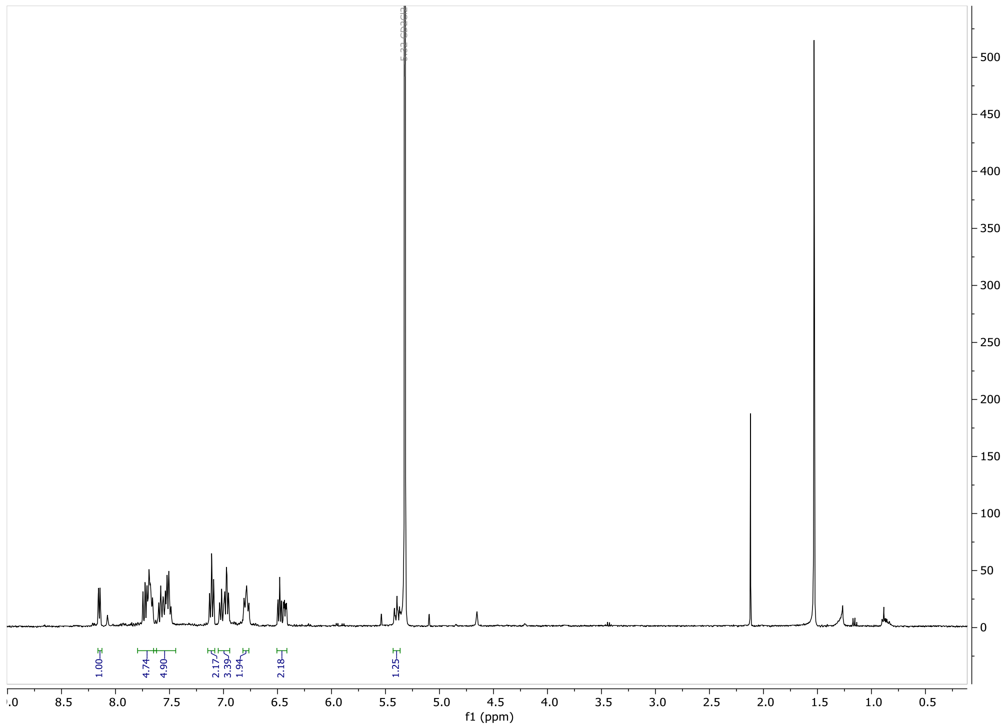

Figure S11. ^{1}H-NMR spectrum of complex **4**.

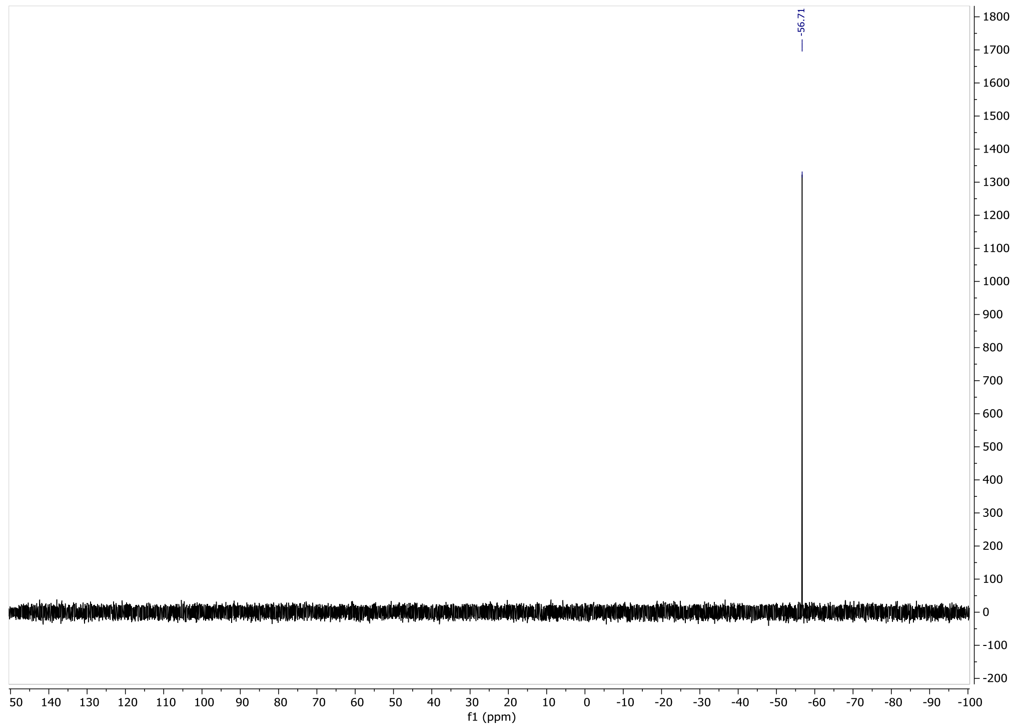

Figure S12. ^{31}P-NMR spectrum of complex **4**.

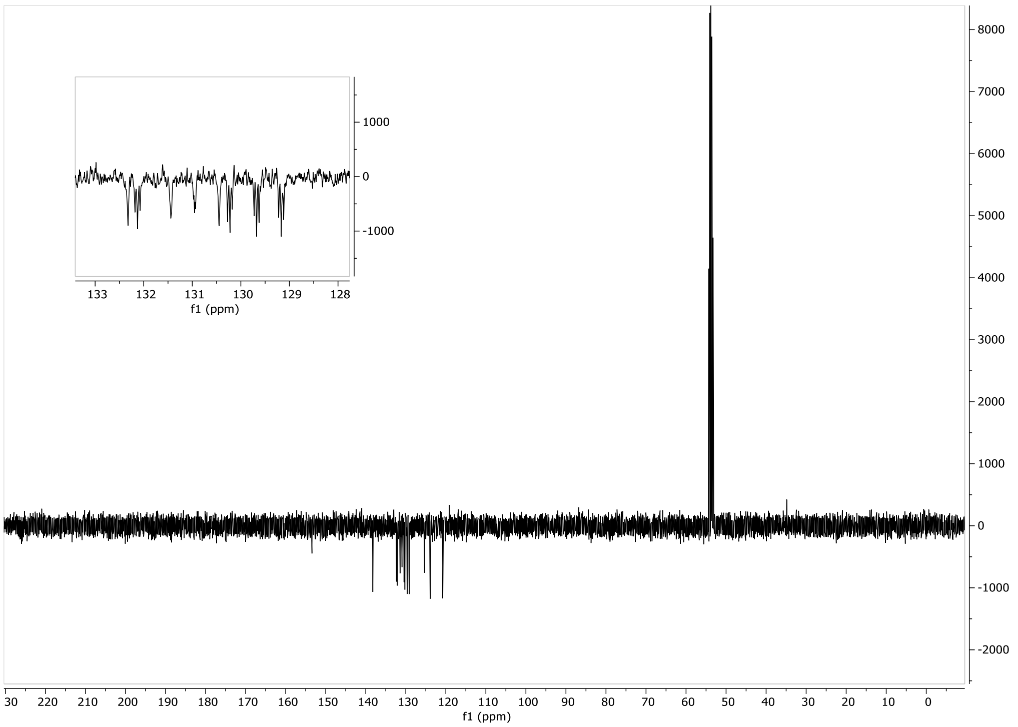

Figure S13. APT-NMR spectrum of complex **4**.

Mass spectra of compounds 1, 2 and 4

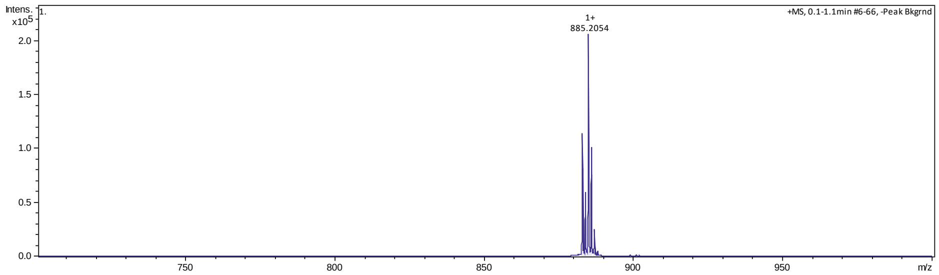

Figure S14. ESI-HRMS spectrum of complex **1**.

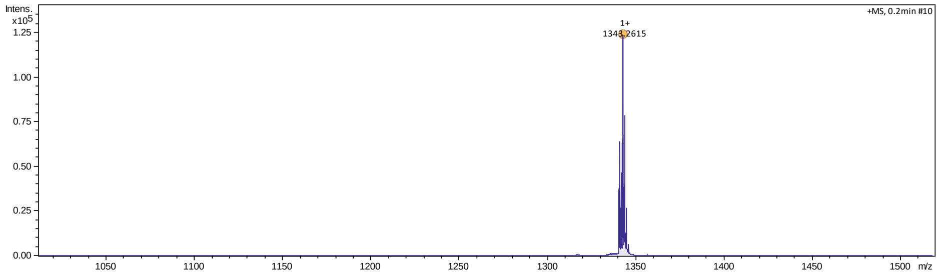

Figure S15. ESI-HRMS spectrum of complex **2**.

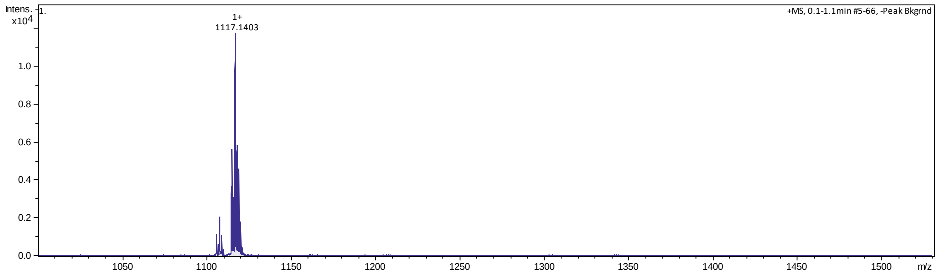

Figure S16. ESI-HRMS spectrum of complex **4**.

Table S1. X-ray data for complex **2.**

Compound	**2**
Formula	C139 H172 Au2 Cl9 F12 Ir2 N4 P8
M_r	3471.94
Crystal size (mm)	0.500 x 0.098 x 0.050
Crystal system	triclinic
Space group	P-1
Cell constants:	
a (Å)	16.1643(17)
b (Å)	17.7210(17)
c (Å)	23.838(3)
α (°)	101.295(2)
β (°)	90.331(2)
γ (°)	100.056(2)
V (Å^3)	6587.6(12)
Z	2
Dx (Mg m^{-3})	1.750
μ(mm^{-1})	4.584
F(000)	3454
T (K)	120
$2\theta_{max}$	51
No. of refl.:	
Measured	91886
Independent	25861
Transmissions	0.5379 and 0.7640
R_{int}	0.0640
Parameters	1531
Restraints	269
Goodness of fit on F^2	1.013
$wR(F^2$, all Refl.)	0.1077
$R(I > 2\sigma(I))$	0.0423
max. Δρ (e Å^{-3})	3.044

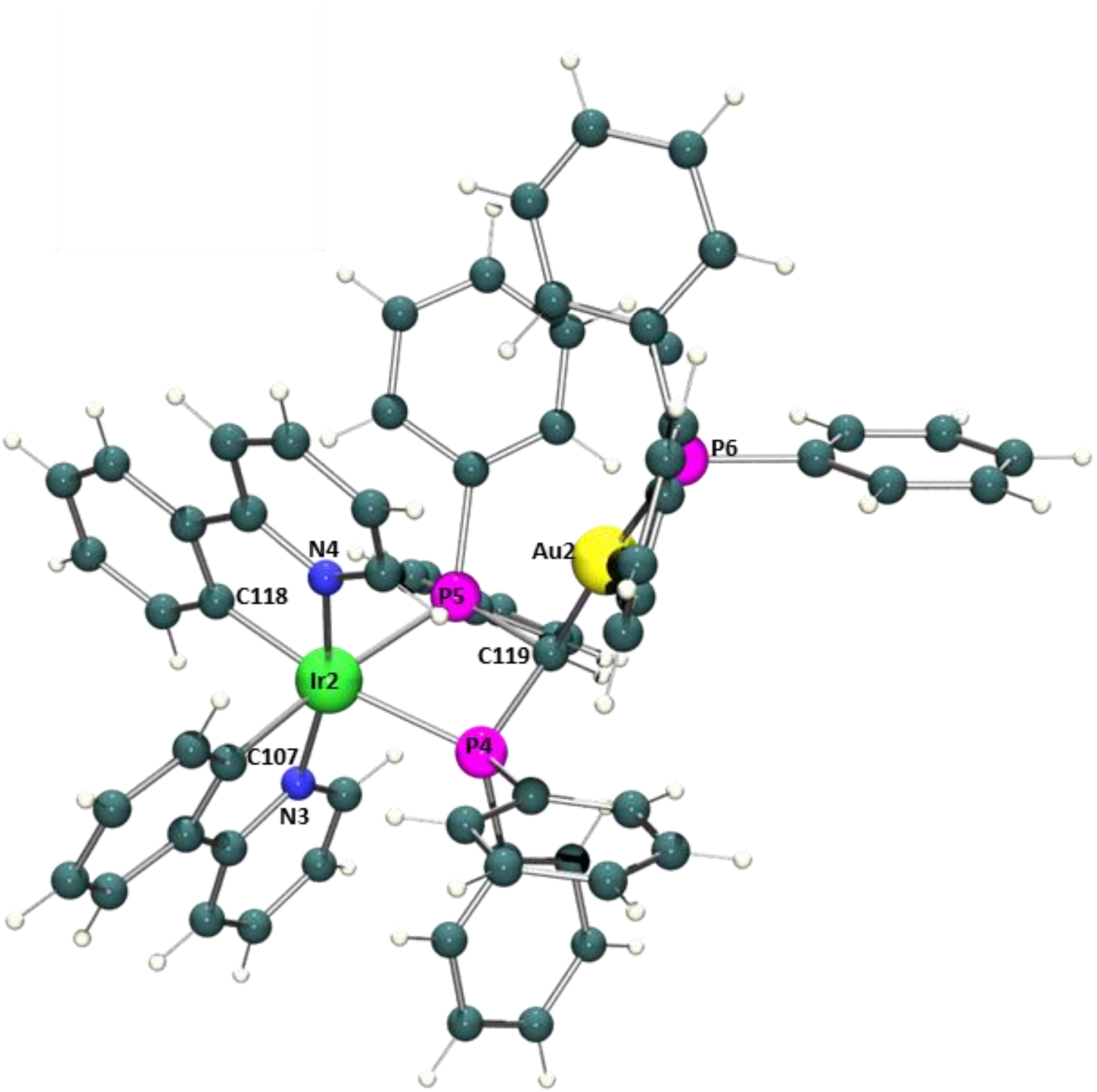

Figure S17. Pov-ray representations of molecule Ir(2) crystallised from complex **2**. Counterion and solvent molecules have been omitted for clarity. The most relevant bond lengths (Å) and angles (DEG): Ir2-C77: 2.062(8), Ir2-C88: 2.053(7), Ir2-N3: 2.080(7), Ir2-N4: 2.060(7), Ir2-P4: 2.382(2), Ir2-P5: 2.414(2), Au2-C84: 2.114(7), Au2-P6: 2.271(2), C84-Au2-P6: 177.7(2), P4-Ir2-P5: 68.94(7), N3-Ir2-N4: 166.1(3).

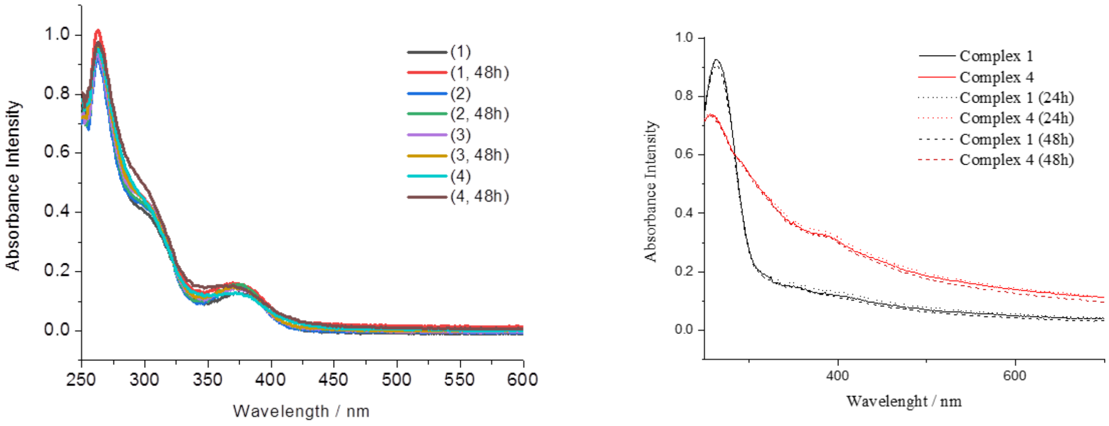

Figure S18. Absorption spectra of complexes **1-4** in DMSO solution and stability studies at 48 hours (left). Stability study of complexes **1** and **4** in a mixture of PBS pH 7.4 and DMSO solution (≲ 5%) and at 48 hours (right).

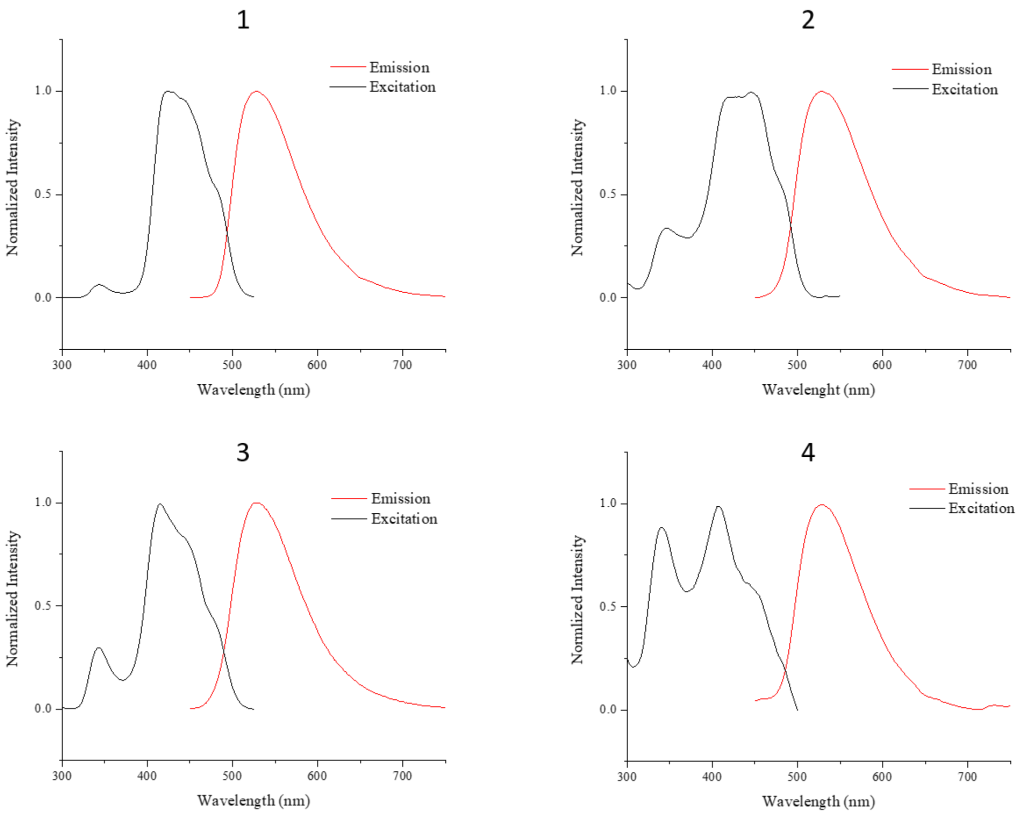

Figure S19 Emission-excitation spectra of complexes **1-4** measured in DSMO solution.

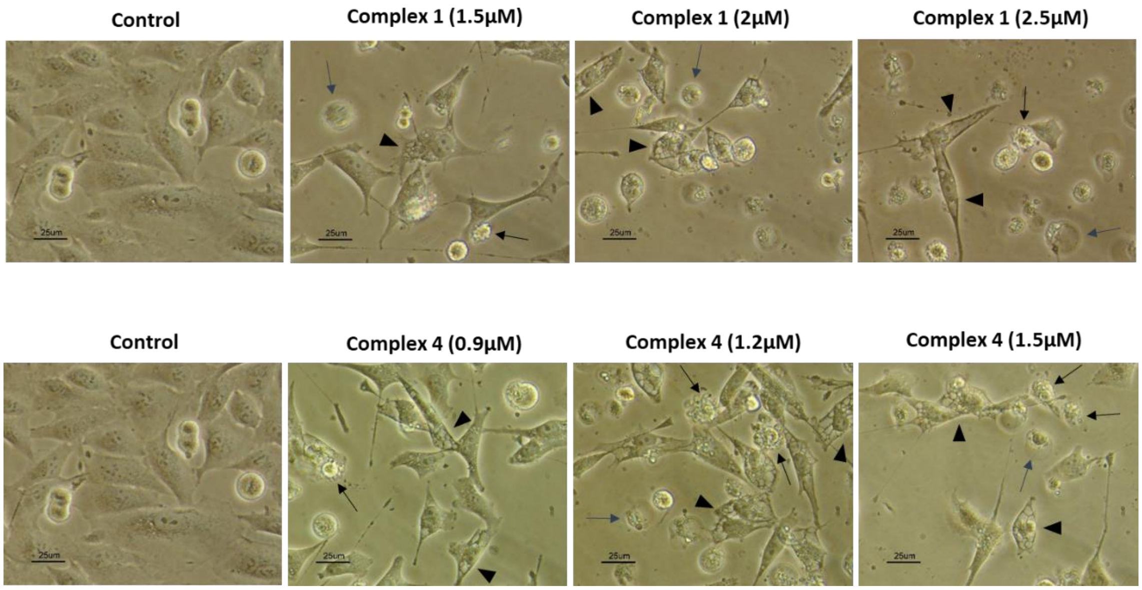

Fig. S20. Phase contrast microscopy images of A549 cells treated with **1** and **4** at different concentrations for 48 h. Black and blue arrows point to examples of apoptotic and necrotic cells, respectively. Little black triangles show cells containing cytoplasmic vacuoles.

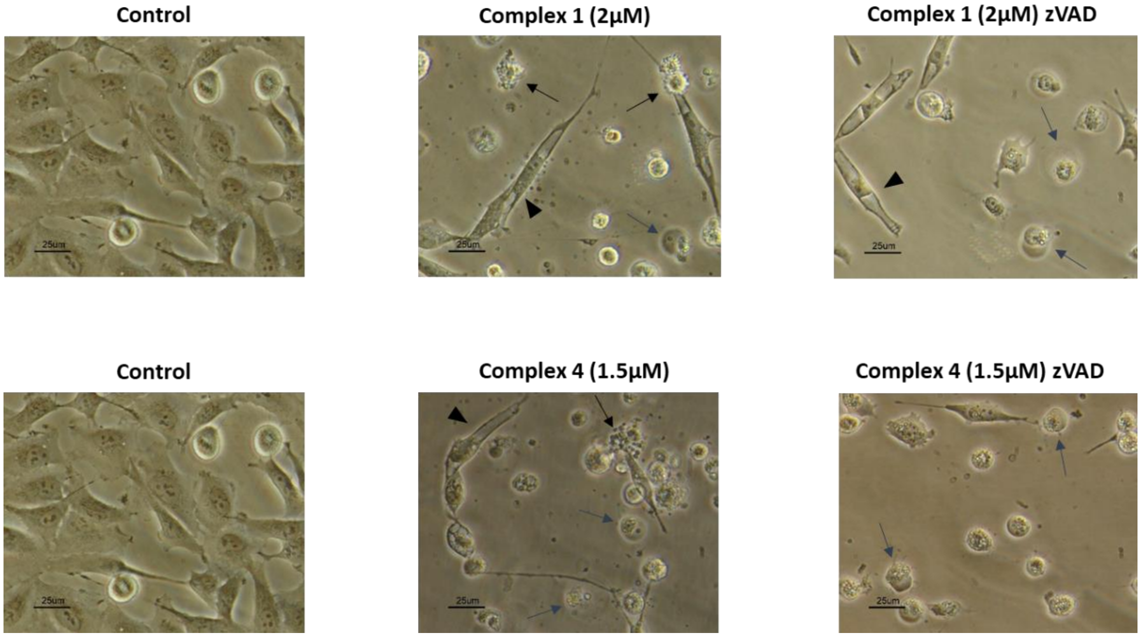

Fig. S21. Phase contrast microscopy images of A549 cells preincubated with Z-VAD (50 μM) and treated with **1** (2 μM) and **4** (1.5 μM) for 48 h. Black and blue arrows point to examples of apoptotic and necrotic cells, respectively. Little black triangles show cells containing cytoplasmic vacuoles.

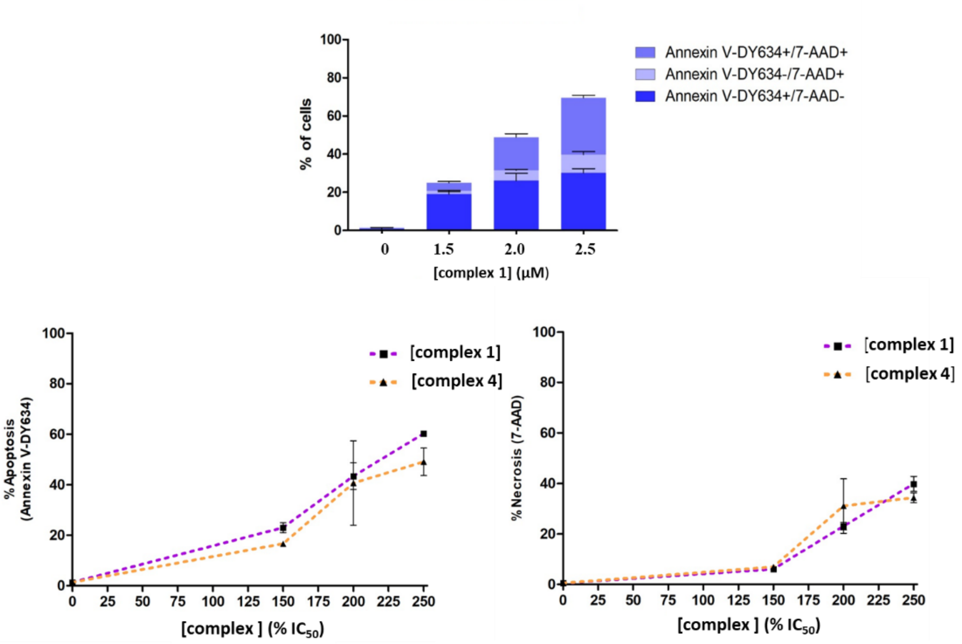

Figure S22. Cell death mechanism induced by complexes **1** and **4** in A549 cells. Top graph: cytotoxic effect of compound **1** evaluated with Annexin V-DY634 and 7-AAD staining. Bottom graphs: percentage of Annexin V-DY634-only (left) or 7-AAD-only (right) labelled cells after exposition to complexes **1** and **4**.

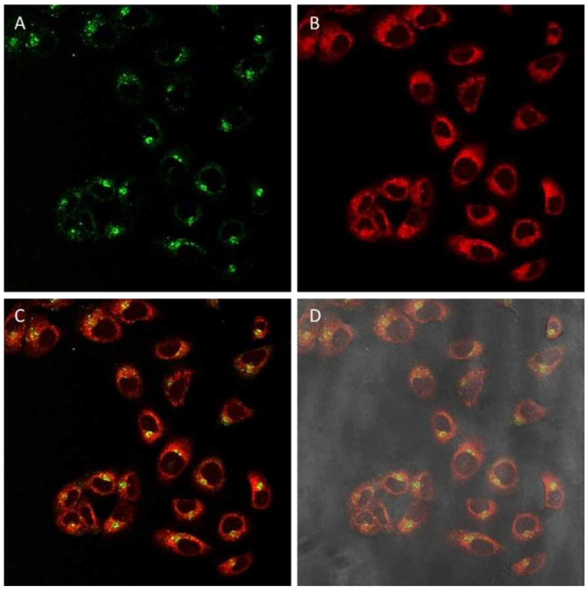

Figure S23. Fluorescence confocal microscopy images of A549 cells incubated with **4** (24 h) and stained with MTR. (A) Image after irradiation at 473 nm; (B) after irradiation at 598 nm; (C) superimposition picture of A, B; (D) superimposition with bright field image. Green: complex **4**, Red: MTR. Images width (↔) 210 μm.

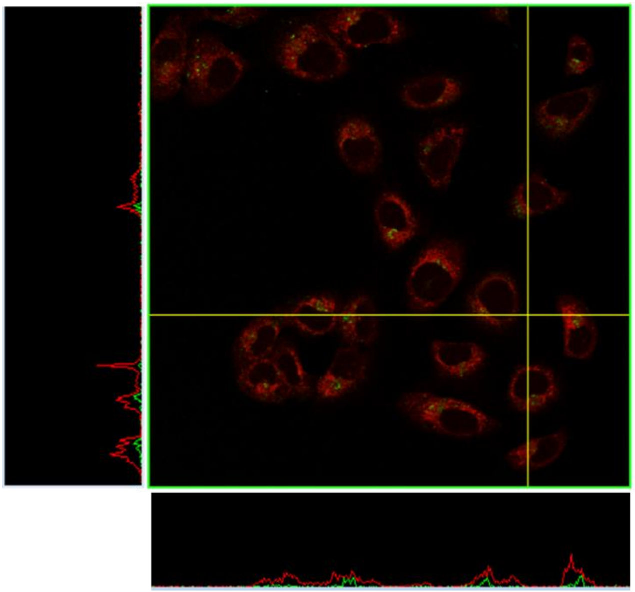

Figure S24. Cross section of intensity for the superimposition image of compound **4** (green, irradiation at 473 nm) with MTR (red, irradiation at 598 nm) incubated in A549 cells. Images width (↔) 210 μm.

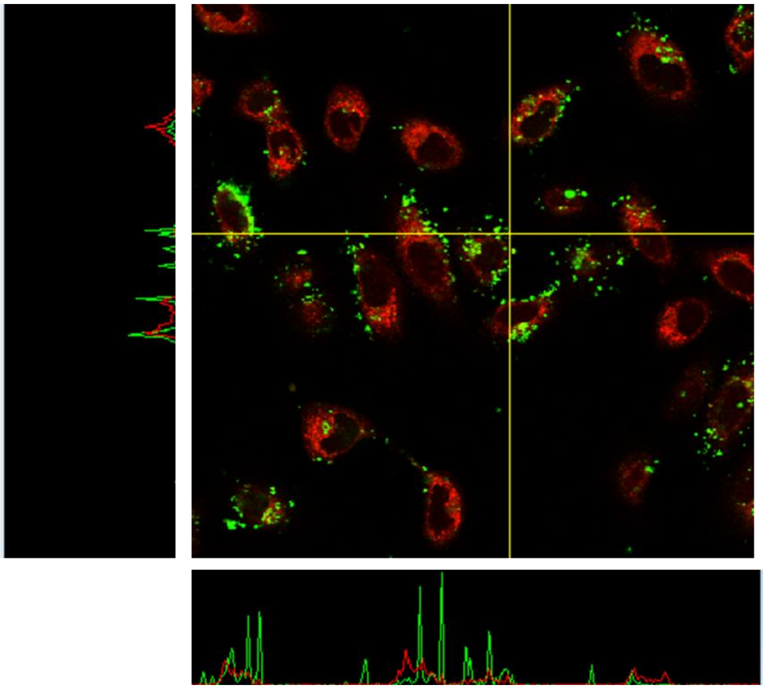

Figure S25. Cross section of intensity for the superimposition image of compound **2** (green, irradiation at 473 nm) with MTR (red, irradiation at 598 nm) incubated in A549 cells. Images width (↔) 210 μm.

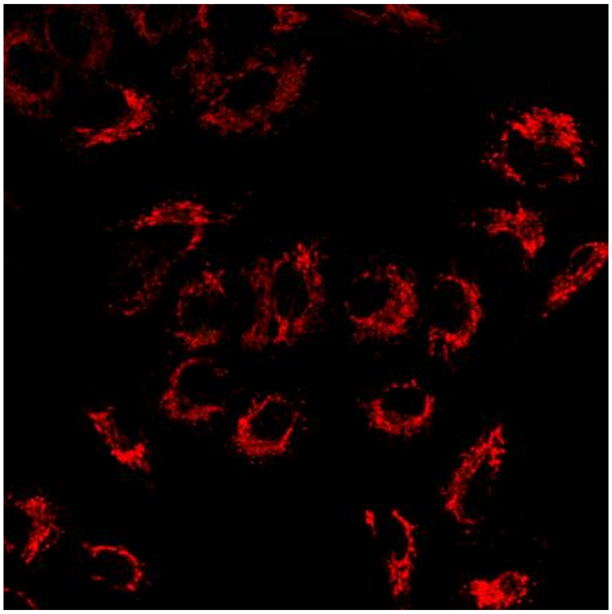

Figure S26. Control cells incubated only with MTR (irradiation at 598 nm). Images width (↔) 210 μm.

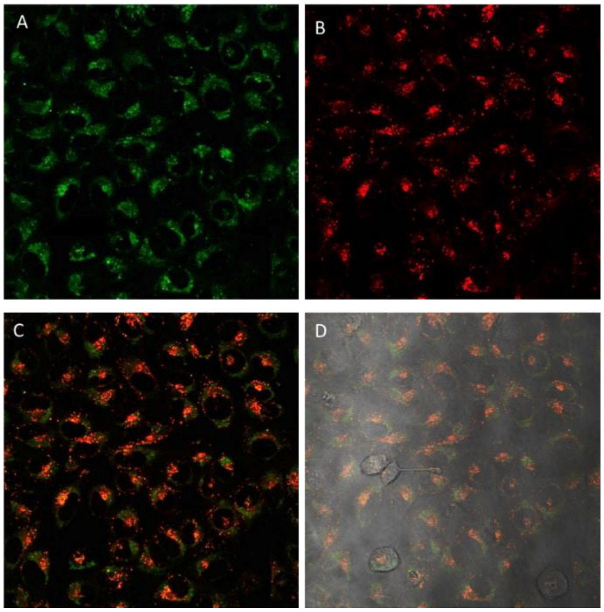

Figure S27. Fluorescence confocal microscopy images of A549 cells incubated with **4** (18 h) and stained with LTR. (A) Image after irradiation at 473 nm; (B) after irradiation at 598 nm; (C) superimposition picture of A, B; (D) superimposition with bright field image. Green: complex **4**, Red: LTR. Images width (↔) 210 μm

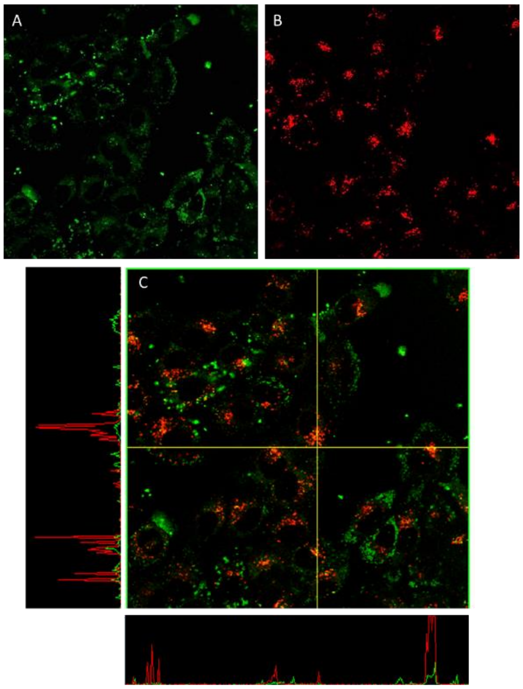

Figure S28. Fluorescence confocal microscopy images of A549 cells incubated with **2** (18 h) and stained with LTR. (A) Image after irradiation at 473 nm; (B) after irradiation at 598 nm; (C) superimposition of pictures A, B and cross-section of their intensities. Green: complex **2**, Red: LTR. Images width (↔) 210 µm.

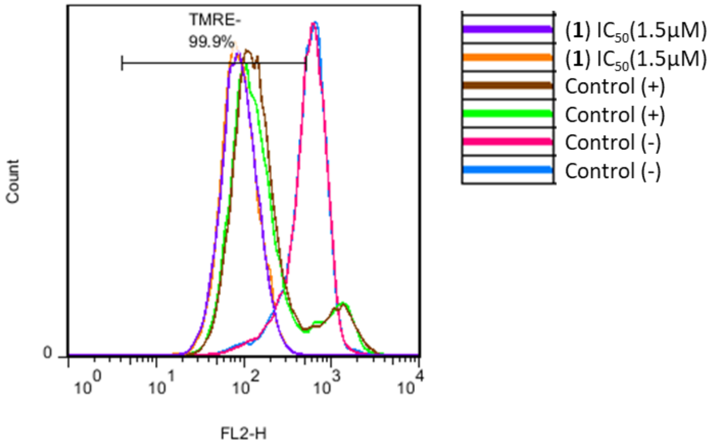

Figure S29. Disruption of mitochondrial transmembrane potential induced by **1** in A549 cells. Positive control S63845.

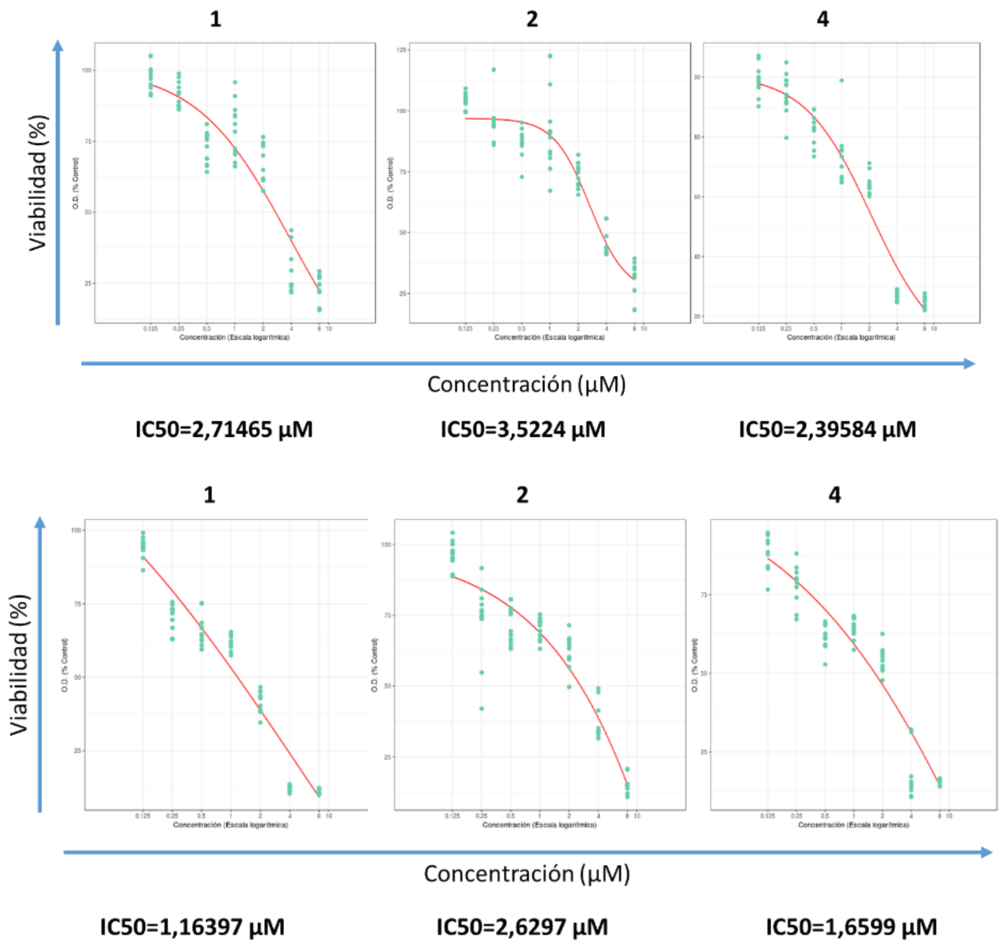

Figure S30. Cell viability of apb-ECFCs (top graph) and ucb-ECFCs (bottom graph) in presence of complexes **1**, **2** and **4**.

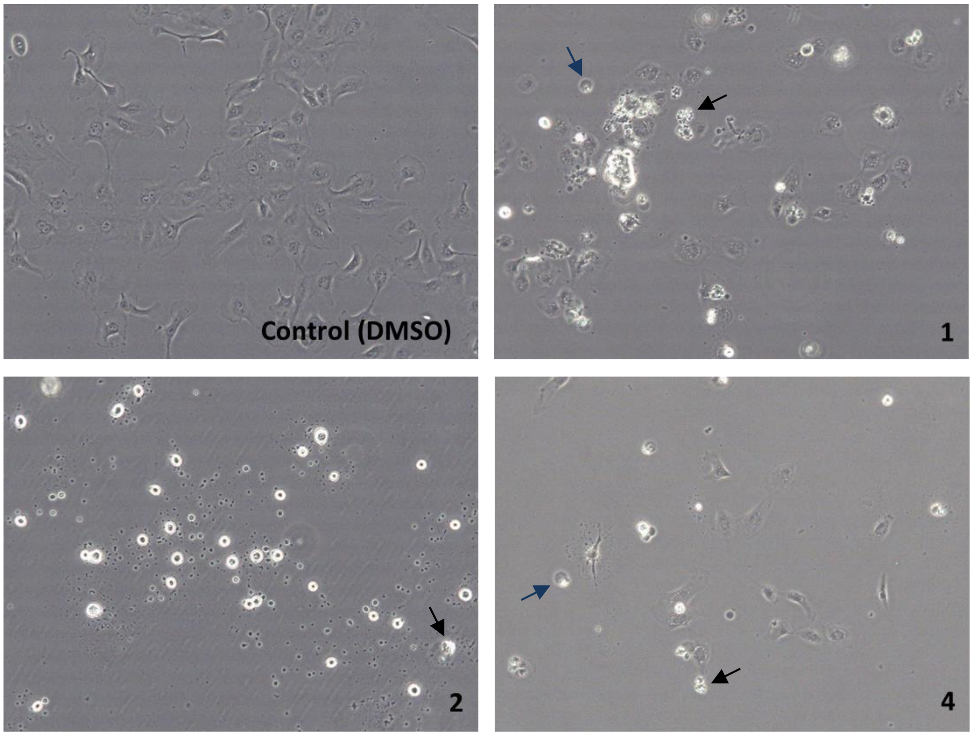

Figure S31. Phase contrast microscopy images of ucb-ECFCs incubated with **1**, **2** and **4** at 1 μM for 24 h. Black and blue arrows point to examples of apoptotic and necrotic cells, respectively.

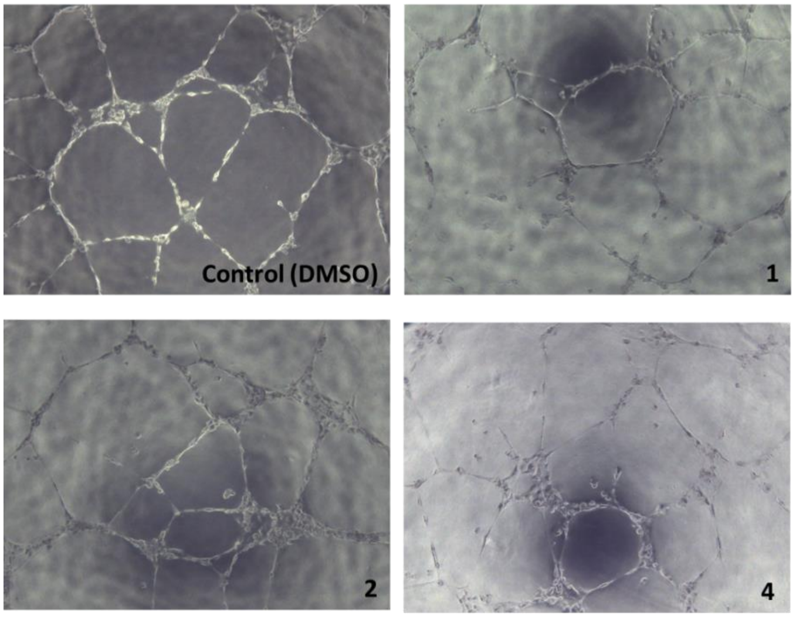

Figure S32. Phase contrast microscopy images of the *in vitro* angiogenic activity assays in apb-ECFCs cells incubated with **1**, **2** and **4** at 0.25 μM for 20 h.
